# PBX1 Genomic Pioneer Function Drives ERα Signaling Underlying Progression in Breast Cancer

**DOI:** 10.1371/journal.pgen.1002368

**Published:** 2011-11-17

**Authors:** Luca Magnani, Elizabeth B. Ballantyne, Xiaoyang Zhang, Mathieu Lupien

**Affiliations:** 1 Norris Cotton Cancer Center, Dartmouth Medical School, Lebanon, New Hampshire, United States of America; 2 Institute of Quantitative Biomedical Sciences, Norris Cotton Cancer Center, Dartmouth Medical School, Lebanon, New Hampshire, United States of America; Fred Hutchinson Cancer Research Center, United States of America

## Abstract

Altered transcriptional programs are a hallmark of diseases, yet how these are established is still ill-defined. PBX1 is a TALE homeodomain protein involved in the development of different types of cancers. The estrogen receptor alpha (ERα) is central to the development of two-thirds of all breast cancers. Here we demonstrate that PBX1 acts as a pioneer factor and is essential for the ERα-mediated transcriptional response driving aggressive tumors in breast cancer. Indeed, PBX1 expression correlates with ERα in primary breast tumors, and breast cancer cells depleted of PBX1 no longer proliferate following estrogen stimulation. Profiling PBX1 recruitment and chromatin accessibility across the genome of breast cancer cells through ChIP-seq and FAIRE-seq reveals that PBX1 is loaded and promotes chromatin openness at specific genomic locations through its capacity to read specific epigenetic signatures. Accordingly, PBX1 guides ERα recruitment to a specific subset of sites. Expression profiling studies demonstrate that PBX1 controls over 70% of the estrogen response. More importantly, the PBX1-dependent transcriptional program is associated with poor-outcome in breast cancer patients. Correspondingly, PBX1 expression alone can discriminate a priori the outcome in ERα-positive breast cancer patients. These features are markedly different from the previously characterized ERα-associated pioneer factor FoxA1. Indeed, PBX1 is the only pioneer factor identified to date that discriminates outcome such as metastasis in ERα-positive breast cancer patients. Together our results reveal that PBX1 is a novel pioneer factor defining aggressive ERα-positive breast tumors, as it guides ERα genomic activity to unique genomic regions promoting a transcriptional program favorable to breast cancer progression.

## Author Summary

Approximately two-thirds of breast cancers depend on the estrogen receptor alpha (ERα) for their growth. Its capacity to act as a transcription factor binding DNA following estrogen stimulation is central to promote a pro-tumorigenic transcriptional response. Importantly, different classes of ERα-positive breast tumors can be discriminated based on outcome. However, the underlying mechanisms driving these differences are unknown. Here we demonstrate that PBX1 acts as a pioneer factor recognizing a specific epigenetic modification to remodel chromatin and guide ERα genomic activity. This translates in a specific transcriptional program associated with poor-outcome in breast cancer patients. Even more, PBX1 expression alone is sufficient to identify a priori ERα-positive breast cancer patients at risk of developing metastasis. Overall, this study defines the mechanisms dependent on the pioneer factor PBX1 that drives an aggressive response in a subset of ERα-positive breast cancers. These features highlight the uniqueness of PBX1 and demonstrate its potential prognostic value.

## Introduction

The implementation of transcriptional programs is central to the commitment of pluripotent cells occurring throughout development [1], [2]. Likewise, diseases commonly arise from altered transcriptional programs. This requires active reprogramming characterized by chromatin remodeling and altered epigenetic signature at lineage-specific functional genomic elements [2]–[5]. The estrogen receptor alpha (ERα) is a nuclear receptor central to breast cancer development. Upon estrogen stimulation, it binds at thousand of genomic loci defining its cistrome to promote a pro-proliferative transcriptional program [6]–[9]. Its genomic actions are in part dependent on the pioneer factor FoxA1 [6], [7], [8], [10], [11], [12], [13], [14]. Pioneer factors are an emerging class of DNA binding proteins. They play a central role in defining transcriptional programs as they can integrate and remodel condensed chromatin rendering it competent for transcription factor binding [6], [15], [16], [17], [18], [19]. Their recruitment to the chromatin is sequence specific and can be facilitated by an epigenetic signature dependent on histone methylation [6], [20].

PBX1 (Pre-B-cell leukemia homeobox 1) is a member of the Three Amino acid Loop Extension (TALE)-class homeodomain family required for diverse developmental processes including hematopoiesis [21], skeleton patterning [22], pancreas [23], and urogenital systems organogenesis [24], [25]. While it is best known as an oncoprotein when fused to E2A in pre-B-cell leukemia [26], it also contributes to prostate, ovarian and esophageal cancer [27]–[30]. It is also highly expressed in breast cancer [31]. PBX1 is a cofactor for homeobox (HOX) transcription factors as it increases their affinity and specificity to chromatin [32], [33]. However, recent interactome studies have revealed that 12% of PBX1 putative partners are non-homeodomain transcription factors [34], [35]. In agreement, PBX1 modulates the transcriptional activity of nuclear receptors such as the thyroid and glucocorticoid receptors and was recently proposed to act as a pioneer factor for the bHLH factor MyoD [36]–[38]. However, the contribution of PBX1 to chromatin structure and epigenetic signatures regulating transcription in ERα-positive breast cancer cells is unknown. In the present study, we have investigated the pioneer function of PBX1 towards ERα genomic activity in breast cancer.

## Results

### PBX1 is essential to the estrogen response in ERα-positive breast cancer cells

**Figure 1:**
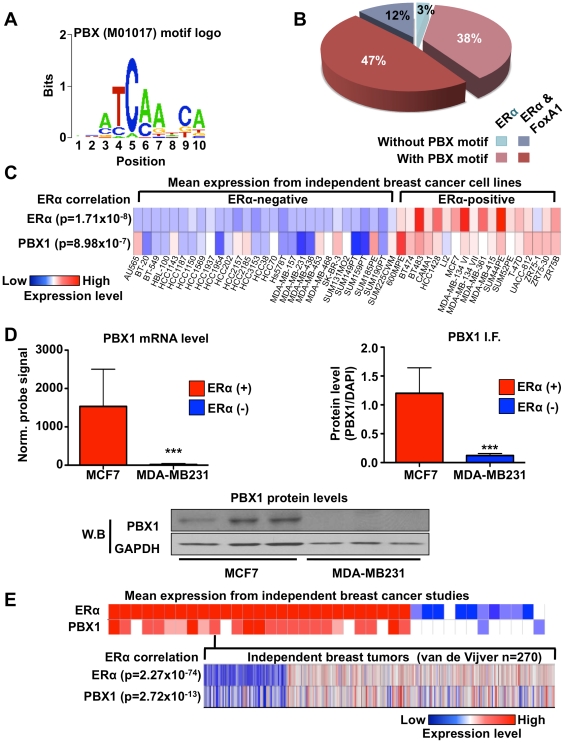
PBX1 correlates with ERα. (A) Motif/sequence logo representation of the PBX1 matrix (Transfac: M01017). (B) The proportion of ERα binding sites harboring the PBX1 matrix (Transfac: M01017) is presented taking into account the overlap of ERα binding sites with FoxA1 binding sites. Percentages are calculated based on the 5782 ERα binding sites. (C) Co-expression of PBX1 and ERα mRNA transcripts is demonstrated across 47 distinct breast cancer cell lines separated based on their ERα-histological status. The p value revealing significant correlation between ERα-histological status and mRNA expression for ERα and PBX1 is presented. (D) Both mRNA (left panel) and protein (derived from immuno-fluorescence or western blot, right and bottom panel respectively) levels for PBX1 correlate with ERα expression status when assessed in ERα-positive (MCF7) and ERα–negative (MDA-MB231) breast cancer cells (average from three independent probes against PBX1 is presented for the mRNA expression analysis provided by bioGPS.org). (E) PBX1 and ERα are co-expressed in primary breast tumors. Expression profiles from primary breast tumors reveals that PBX1 mRNA levels are correlated with ERα-histological status and ERα mRNA expression in primary breast tumors (meta-analysis conducted using Oncomine). (*<0.05, **<0.01***, <0.001 p value).

Condensed chromatin constitutes a barrier for the recruitment of transcription factors to the DNA. FoxA1 binding at specific genomic regions allows for chromatin remodeling favorable to ERα recruitment at a subset of its cistrome [6], [8], [13], [19], [39]. However, ERα is recruited to thousands of FoxA1-independent sites across the genome [6]. To identify candidate pioneer factors guiding ERα recruitment to the chromatin at these sites we performed seeded motif analyzes using the Cistrome-web application (http://cistrome.dfci.harvard.edu/ap/). This revealed that over 85% of the ERα cistrome harbors the DNA motif recognized by PBX1 (Figure 1A and 1B). Noteworthy, the presence of the PBX1 motif in ERα binding sites was significantly different from another similar size cistrome (androgen receptor (AR) cistrome from LNCaP cells, p<1e-99) (Figure S1A).

Analyzing expression profiles from the NCI60 panel of cancer cells compiled on bioGPS (http://biogps.gnf.org) [40], [41] reveals that PBX1 is significantly co-expressed with ERα (co-expression coefficient 0.7784 using probe 205253_at) (Table S1). This was also revealed by comparing PBX1 mRNA expression across 47 distinct ERα-positive and negative breast cancer cells (p = 8.98e-7) (Figure 1C). ERα mRNA expression was also significantly correlated with ERα-histological status of breast cancer cells (p = 1.71e-8) (Figure 1C). These results are further supported by RT-qPCR, immunofluorescence and western blot analyzes in ERα-positive MCF7 and ERα-negative MDA-MB231 breast cancer cells demonstrating co-expression of ERα and PBX1 at the mRNA and protein level (Figure 1D). PBX1 is one of four PBX family members [33]. RT-qPCR against other PBX1 genes demonstrates that PBX1 is the predominant family member expressed in ERα-positive breast cancer cells (Figure S1B). Analyses of 41 independent breast cancer expression profile studies, such as van de Vijver study, demonstrate that PBX1 and ERα are also co-expressed in primary breast tumors (p = 2.72e-13 for the van De Vijver study and p≤1e-4 for all other studies) (Figure 1E) [42]. The correlation between ERα mRNA expression and ERα-histological status is also reported for the van de Vijver study (p = 2.27e-74) (Figure 1E).

**Figure 2:**
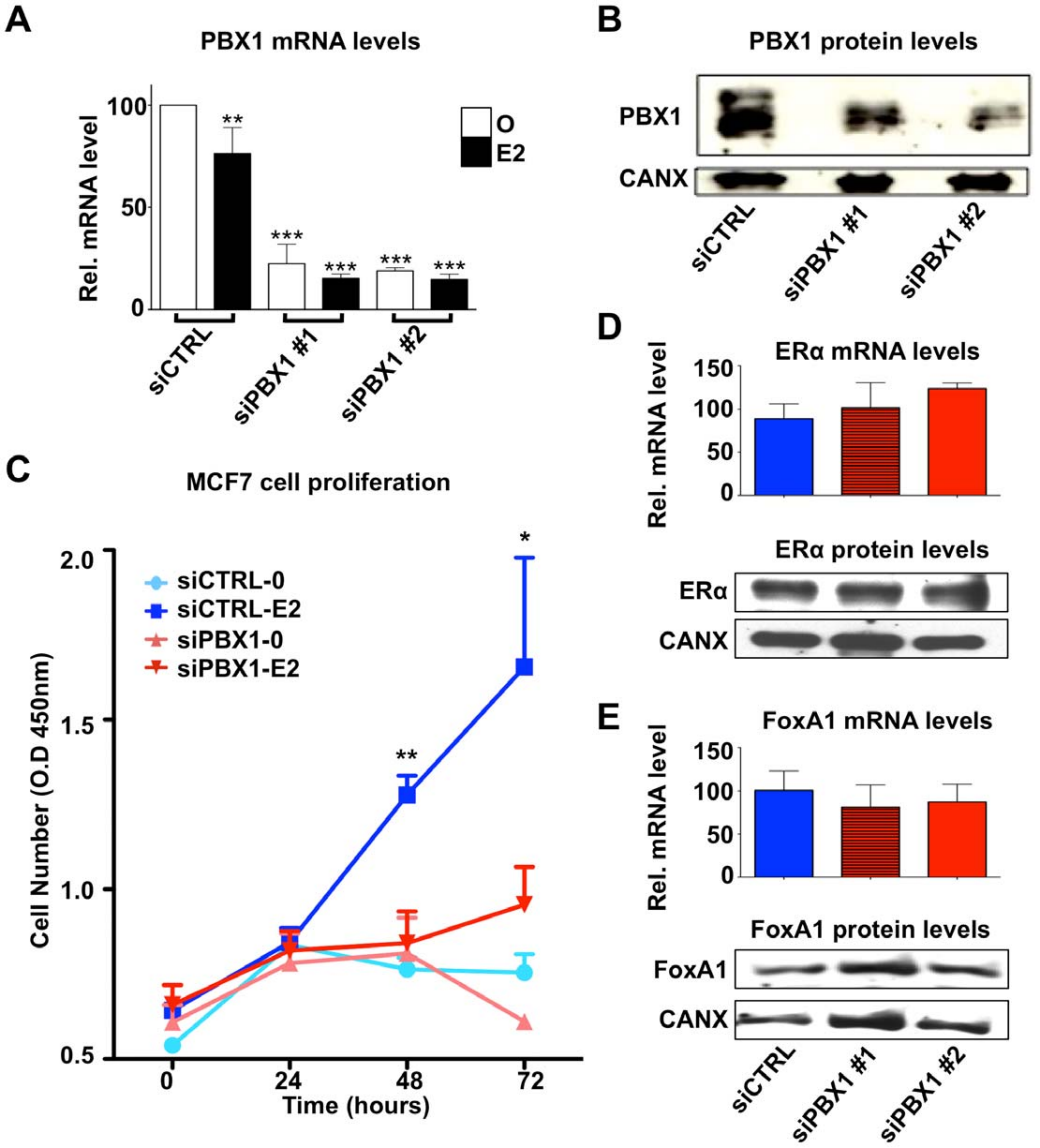
PBX1 is required for the estrogen response in MCF7 cells. (A) PBX1 depletion via siRNA effectively reduces its mRNA and (B) protein levels. (C) MCF7 breast cancer cells depleted of PBX1 fail to proliferate in response to estrogen/17β-estradiol (E2) stimulation compared to control treatment (O). (D) PBX1 silencing does not alter ERα or FoxA1 mRNA (histogram) or protein levels (Western Blot, WB). (*<0.05, **<0.01***, <0.001 p value).

To address the functional relation between PBX1 and ERα we assessed the role of PBX1 on estrogen-induced growth in the ERα-positive MCF7 breast cancer cells. PBX1 mRNA and protein levels were significantly depleted (∼70%) in MCF7 breast cancer cells transfected with one of two independent siRNA against PBX1 (Figure 2A and 2B). In agreement with a role for PBX1 in breast cancer [27], PBX1 depletion completely prevented the estrogen-induced proliferation of MCF7 breast cancer cells (Figure 2C and S2A-B). Importantly, PBX1 depletion in MCF7 breast cancer cells did not affect ERα or FoxA1 expression both at the mRNA and protein level (Figure 2D). Overall these results support a functional role for PBX1 in mediating the response to estrogen in ERα-positive breast cancer.

### PBX1 marks functional ERα binding sites

**Figure 3:**
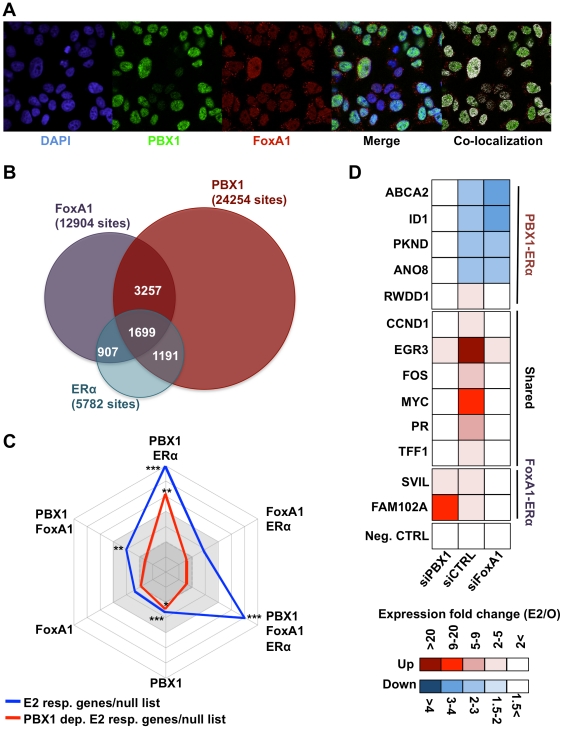
PBX1 marks functional ERα bindings. (A) Confocal immunofluorescence analysis in MCF7 cells cultured in absence of estrogen/17β-estradiol (E2) reveals that PBX1 is localized in the nucleus of MCF7 breast cancer cells and partially overlap with the pioneer factor FoxA1. (B) Venn diagram of PBX1 (Full media), ERα (after estrogen stimulation) and FoxA1 (full media) cistromes reveal their significant overlap on the chromatin. (C) A comparison between E2 responsive genes (all or PBX1-dependent) and the unique versus shared ERα, FoxA1 and PBX1 binding sites defined in Figure 3B was performed by normalizing the number of responsive genes with at least one unique or shared binding site a given factor within ±20 kb of their transcription start site (TSS) to the number of unresponsive genes with at least one binding sites from the same type of site within ±20 kb of their TSS. The values for all E2 responsive genes (blue line) and PBX1-dependent E2 responsive genes (red line) were plotted in a radar format (1< dark grey area, 1–2 light grey area, >2 white area, ticks are 0.5 increments). (*<0.01, **<0.001***, <0.00001 p value). (D) RT-qPCR against E2 target genes associated with PBX1-FoxA1-ERα, PBX1-ERα or FoxA1-ERα binding sites based on Figure 3C was performed in MCF7 breast cancer cells depleted of PBX1 (siPBX1) or Foxa1 (siFoxA1). A control siRNA (siCTRL) was used for comparison.

Estrogen signaling involves ERα activation and subsequent recruitment to the chromatin. Pioneer factors can therefore be identified through their role at the chromatin prior to estrogen treatment. Immunofluorescence assays against PBX1 in MCF7 breast cancer cells deprived of estrogen demonstrate its localization to the nucleus (Figure 3A). While PBX1 and FoxA1 have a similar nuclear distribution, confocal immunofluorescence analysis against FoxA1 reveals that it only partially overlaps with PBX1 (Figure 3A and Figure S3A and S3B). To demonstrate that PBX1 occupies the chromatin in MCF7 breast cancer cells we performed a ChIP-seq assay in cells maintained in full media. This identified 24254 high-confidence PBX1 sites (p≤1e-5) predominantly localized a distant regulatory elements (Figure 3B and Figures S4A and S4B, S5, S6, S7, S8). Directed ChIP-qPCR assays on 37 randomly selected PBX1 bound sites identified by ChIP-seq demonstrates that it is loaded to the chromatin in absence of estrogen (Figure S4B). Approximately 50% of the estrogen-induced ERα cistrome overlaps with PBX1 bound sites (Figure 3B). A significant overlap between ERα and PBX1 is also observed for all publically available ERα cistromes (Figure S9) [6], [7], [9], [43], [44], [45], [46], [47], [48], [49], [50], [51]. FoxA1 is loaded to the majority of these sites (Figure 3B). In fact, ChIP-reChIP assays in MCF7 breast cancer cells maintained in estrogen free media demonstrates that both pioneer factors co-localize on the chromatin at shared sites (Figure S11). Importantly, over 37% of the FoxA1-independent ERα binding sites overlap with PBX1 (Figure 3B). Expression profile analysis in MCF7 breast cancer depleted of PBX1 reveals that a 71% of estrogen-induced target genes are dependent on PBX1 (Table S2 and Figure S12). Importantly, the estrogen signature identified by this expression profile was highly enriched for genes defining ERα-positive primary breast tumors (p = 5.75e-10) [52].

To assess the relation between genome-wide binding and expression profiles we cross-examined the estrogen responsive gene lists (all estrogen responsive genes and PBX1-dependent estrogen responsive genes) defined in MCF7 breast cancer cells against the binding profiles for ERα, PBX1 and FoxA1. This was accomplished by determining the number of estrogen responsive genes (all or PBX1-dependent) harboring at least one binding sites shared or unique to a given factor within ±20 kb from their transcription start site (TSS). This was repeated for the null list consisting of all genes from the refseq gene list not regulated upon estrogen stimulation in MCF7 breast cancer cells. The ratio of estrogen responsive genes associated with binding events within ±20 kb of their TSS over the number of genes from the null list associated with binding events within ±20 kb of their TSS was then plotted in a radar format. Estrogen target genes were significantly associated with PBX1-ERα shared sites (7% of total estrogen-responsive genes) and PBX1-FoxA1-ERα shared sites (12% of total estrogen-responsive genes) (blue line, Figure 3C). FoxA1-ERα shared sites did not preferentially associate with estrogen regulated genes (Figure 3C). Remarkably, PBX1-dependent estrogen target genes were specifically associated with PBX1 unique and PBX1-ERα shared sites (red line, Figure 3C). This was validated through RT-qPCR against estrogen target genes dependent on PBX1, FoxA1 or both. Indeed, PBX1 depletion disrupted only the regulation of shared or PBX1-dependent estrogen target genes in MCF7 breast cancer (Figure 3D and Figure S13). Conversely, FoxA1 silencing impacted only the regulation of shared and FoxA1-dependent estrogen target genes (Figure 3D and Figure S13). Collectively, these data support the notion that PBX1 is required to regulate a specific subset of estrogen responsive genes. Moreover, they suggest that PBX1 is required for the implementation of an estrogen regulated transcriptional program distinct from FoxA1.

### PBX1 controls ERα genomics activity

**Figure 4:**
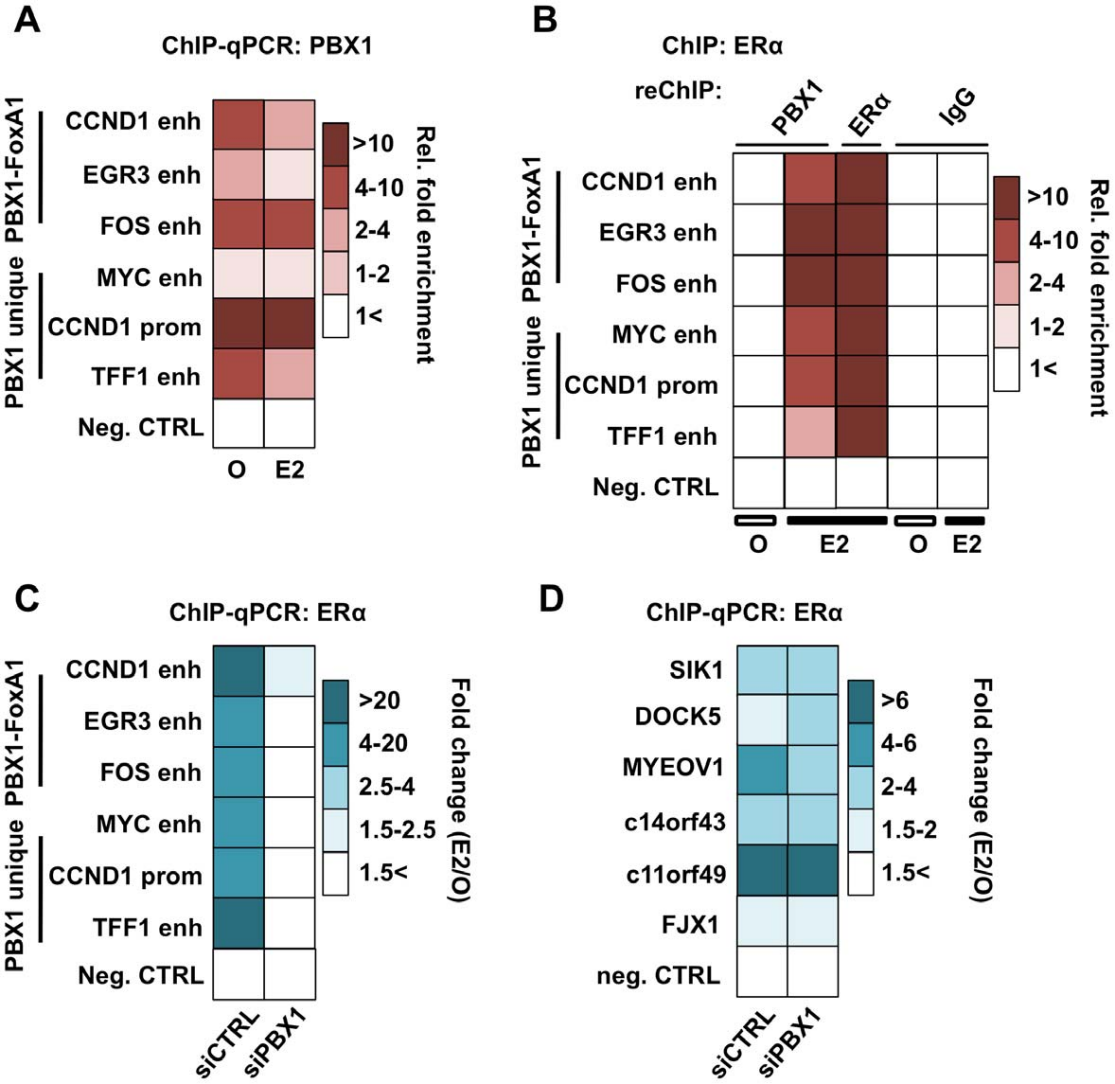
PBX1 is located in the nucleus and mediates ERα genomics activity. (A) PBX1 occupies ERα genomic targets prior to its recruitment following estrogen/17β-estradiol (E2) stimulation compared to control treated cells (O). Similarly, PBX1 remains bound to the chromatin after E2 treatment in MCF7 breast cancer cells as determined by ChIP-qPCR. (B) ChIP-reChIP assays reveal that PBX1 and ERα co-occupy the same genomic regions upon E2 stimulation. In addition to a negative control site, matched IgG were used as a negative control in the reChIP assay. (C) PBX1 silencing (siPBX1) abrogates ERα recruitment at regulatory elements in MCF7 breast cancer cells compared to control (siCTRL). Values are calculated as a ratio between untreated and E2 treated relative fold enrichment defined by ChIP-qPCR. (D) ChIP-qPCR against ERα at PBX1-independent sites demonstrates that ERα recruitment is not disrupted at these sites upon PBX1 silencing. Values are calculated as a ratio between untreated and E2 treated relative fold enrichment.

ERα-dependent transcriptional response is dependent on its recruitment to the chromatin following estrogen stimulation. To test if PBX1 directly impacts ERα genomic activity we first assessed PBX1 occupancy through ChIP-qPCR assays at known ERα binding sites in MCF7 breast cancer cells treated or not with estrogen. Focusing on both FoxA1-dependent and independent ERα binding sites overlapping with PBX1 (Figure S4C), our results demonstrate that PBX1 is pre-loaded on the chromatin prior to estrogen treatment and remains bound following estrogen treatment (Figure 4A). These sites were chosen from our genome-wide analysis since they are proximal to genes fundamental for breast cancer proliferation and ERα biology. For instance, Myc, CCND1, FOS and EGR3 are well-studied ERα targets promoting breast cancer growth and progression [53], [54], [55]. TFF1 (also known as PS2) is the prototypical estrogen target gene [56]. Sequential ChIP assays (ChIP-reChIP) against ERα and PBX1 in both estrogen treated and untreated MCF7 breast cancer cells demonstrates that both factors co-occupy the same sites following ERα recruitment (Figure 4B).

ChIP-qPCR assays against ERα in PBX1 depleted MCF7 breast cancer cells demonstrate that ERα recruitment following estrogen treatment is dependent on PBX1 (Figure 4C). Importantly, ERα recruitment is disrupted selectively at sites with pre-loaded PBX1 but not at PBX1-independent sites (Figure 4D and Figure S4D) thus ruling out the possibility of a widespread non-specific impact on ERα ability to bind DNA in cells depleted of PBX1. Overall these results demonstrate that PBX1 can occupy the chromatin prior to ERα recruitment and is required for its genomic activity driving estrogen target gene expression. This is in agreement with a role for PBX1 as a novel pioneer factor in breast cancer.

### PBX1 actively impart open chromatin structure at regulatory elements

**Figure 5:**
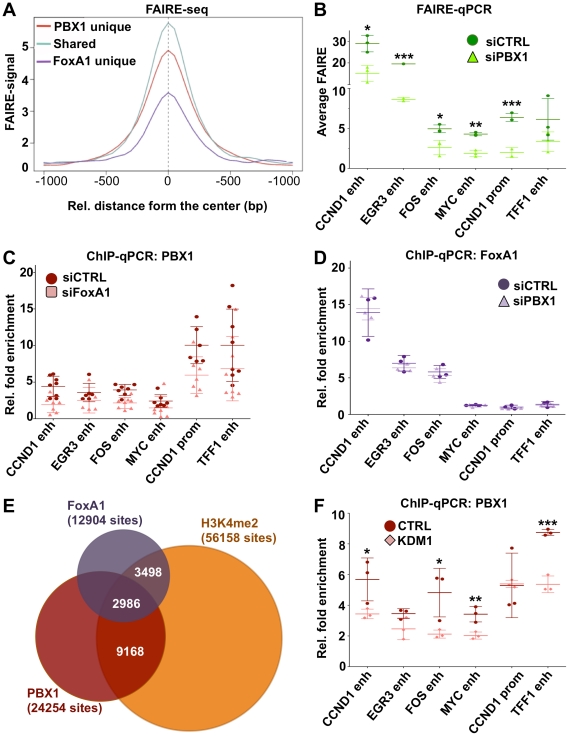
PBX1 is an independent pioneer factor required for chromatin openness whose binding is favored by H3K4me2. (A) Genome wide FAIRE profiles (FAIRE-seq) from MCF7 breast cancer cells maintained in estrogen-free media demonstrate that PBX1 alone or in combination with FoxA1 correlates with open chromatin. (B) Depletion of PBX1 (siPBX1) in MCF7 breast cancer cells maintained in estrogen-free media significantly reduces chromatin openness at PBX1 binding sites compared to control siRNA transfected cells (siCTRL) as measured by FAIRE-qPCR. (C) FoxA1 silencing (siFoxA1) does not alter PBX1 binding to the chromatin compared to control (siCTRL) in MCF7 breast cancer cells maintained in estrogen-free media. (D) PBX1 silencing in MCF7 breast cancer cells maintained in estrogen-free media does not affect FoxA1 binding to the chromatin compared to control. (E) Venn diagram of PBX1 and FoxA1 cistromes defined in full-media as well as H3K4me2 epigenome defined in MCF7 breast cancer cells maintained in estrogen-free media reveals their overlap. (F) Over-expression of the H3K4me2 demethylases KDM1 in MCF7 breast cancer cells maintained in estrogen-free media results in a significant reduction of PBX1 binding to the chromatin compared to the empty vector control (CTRL). (*<0.05, **<0.01***, <0.001 p value).

Chromatin structure inherently represents an obstacle for transcription factor activity. Through their ability to integrate and open condensed chromatin, pioneer factors act as molecular beacons for other transcription factors. Using FAIRE (Formaldehyde Assisted Isolation of Regulatory Elements) assays [39], [57] to measure chromatin condensation/openness prior to estrogen stimulation, we demonstrate that PBX1 acts as a pioneer factor. Indeed, genome-wide FAIRE-seq assays in MCF7 breast cancer cells [44] reveals that PBX1 occupied chromatin is already highly accessible (Figure 5A and Figure S14). Interestingly, the pioneering activity of PBX1 and FoxA1 is synergistic on shared sites (Figure 5A). Sites only bound by FoxA1 are the least accessible (Figure 5A). Comparing FAIRE signal in estrogen starved MCF7 breast cancer cells depleted or not of PBX1 through siRNA revealed a significant decrease in chromatin openness in PBX1-depleted compared to control cells at the majority of tested sites (Figure 5B). In agreement, we demonstrate that PBX1 depletion in MCF7 breast cancer cells seen at the mRNA and protein level (Figure 2A and 2B) also significantly decreases its occupancy on the chromatin (Figure S10B). These results suggest that PBX1 plays a central role in increasing chromatin accessibility essential for transcription factor recruitment further supporting its role as a pioneer factor in breast cancer cells.

Immunofluorescence, ChIP-seq assays and ChIP-reChIP against PBX1 and FoxA1 suggests that they co-occupy genomic regions in MCF7 breast cancer cells (Figure 3A and 3B, Figures S3A and S3B, S9, S10, and S11). To determine if they collaborate with each other at these genomic regions or if they are part of a common complex we profiled FoxA1 binding following PBX1 depletion in estrogen starved MCF7 breast cancer cells. In agreement with both pioneer factors acting independently of each other, FoxA1 depletion did not alter PBX1 binding to the chromatin (Figure 5C). Similarly, PBX1 depletion did not affect FoxA1 recruitment to the chromatin (Figure 5D). Overall, these results reveal that PBX1 acts as a pioneer factor guiding ERα genomic activity independently of FoxA1 in breast cancer.

Covalent modifications are a main staple of epigenetic regulation. Previous reports have demonstrated that methylation of histone H3 on lysine 4 (H3K4me) can define functional regulatory element [58]–[61]. Furthermore, cell type-specific distribution of the mono and di-methylated H3K4 (H3K4me1 and me2) epigenetic modifications are central to cell type-specific transcriptional responses [6], [59], [60]. In cancer cells, depletion of H3K4me2 interferes with FoxA1 binding to chromatin [6], [39]. However, the relationship between FoxA1 and H3K4me2 may not be unidirectional, recent evidence suggesting that FoxA1 can favor H3K4me2 deposition [62]. Genome-wide analysis revealed that H3K4me2 is present on approximately 50% of the PBX1 cistrome (Figure 5E). A similar proportion of FoxA1 cistrome overlaps with the H3K4me2 distribution in MCF7 breast cancer cells (Figure 5E). To test if H3K4me2 favors PBX1 binding to the chromatin we overexpressed H3K4me2 demethylase KDM1 (LSD1/BCH110) and determined PBX1 chromatin occupancy through ChIP-qPCR assays. KDM1 over-expression led to a significant reduction of bound PBX1 in estrogen starved MCF7 cells (Figure 5F). In contrast, PBX1 depletion had no effect on H3K4me2 levels and did not affect KDM1 expression (Figure S15A and S15B). Hence, similarly to FoxA1, the H3K4me2 epigenetic signature favors PBX1 binding.

### PBX1 is a novel prognostic factor that discriminated ERα breast cancer outcomes

**Figure 6:**
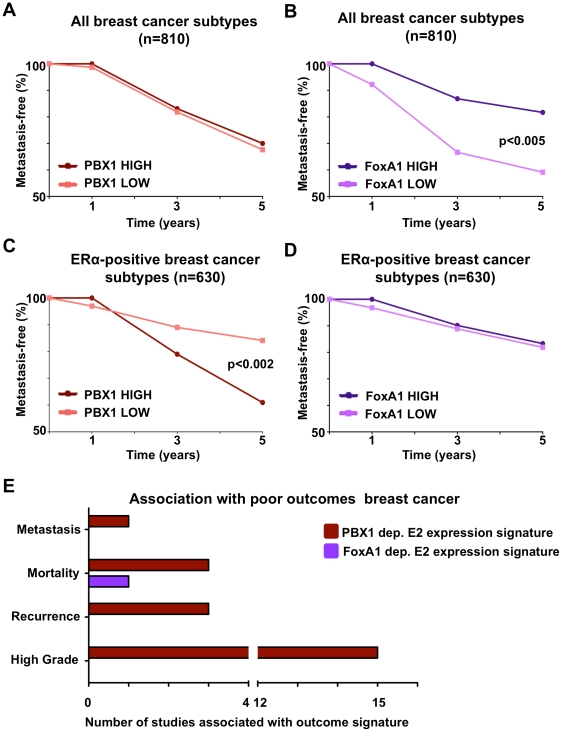
PBX1 is a novel prognostic marker for ERα positive breast cancers. (A–B) PBX1 and FoxA1 prognostic value against metastasis-free survival were investigated against all breast cancer subtypes through Kaplan-Meier curves derived from a meta-analysis of independent expression profile studies from primary breast tumors available through Oncomine. (C–D) The same analysis was repeated while focusing only on the ERα-positive patients. Statistical difference in outcomes between patients with high (top 10% expressing patients) versus low (bottom 10% expressing patients) mRNA level was performed using Fisher exact test. (E) The number of expression signatures associated with poor-outcome defined in primary breast tumors in independent expression profile studies that are significantly associated with PBX1-dependent or FoxA1-dependent estrogen/17β-estradiol (E2) gene signatures is presented (p<0.01, O.R. >2). Results were generated using Oncomine concepts map analysis.

ERα drives proliferation in over 70% of all breast cancers. Accordingly it serves both as a therapeutic target and prognostic factor [63]. In addition, ERα is to date the most exploited marker in the clinic and generally associates with good outcome [64]. FoxA1 does not appear to provide any additional power to discriminate breast cancer subtypes in comparison to ERα profiling [65]–[67]. To assess the prognostic value of PBX1 in breast cancer we performed a meta-analysis using breast tumor expression studies with follow-up data available through Oncomine (Compendia Bioscience, Ann Arbor, MI). We differentiated breast cancer patients according to high (top 10%) or low (bottom 10%) PBX1 mRNA levels and then generated Kaplan-Meier curves according to the metastasis-free survival status of breast cancer patients. In addition, we independently generated Kaplan-Meier curves using the KMplot web application [68]. Results derived from this analysis performed against FoxA1 confirmed previous reports limiting its prognostic value to identify ERα-positive breast cancers within all breast cancer subtypes. PBX1 expression did not discriminate outcome in these same patients (Figure 6A and 6B and B) Interestingly, while FoxA1 mRNA levels where predictive of ERα status, PBX1 levels were evenly distributed in the ERα-positive breast cancer subgroups or all-cases (Figure S17). By focusing our analysis on ERα-positive breast cancer patients (as defined by pathological staining) we revealed the prognostic value of PBX1. Indeed, ERα-positive breast tumors with high PBX1 expression levels are associated with a reduced metastasis-free survival compared to ERα-positive breast tumors with low PBX1 expression (p<0.002) (Figure 6C and Figure S16C and S16D). FoxA1 expression could not stratify metastasis-free survival within ERα-positive breast cancer patients (Figure 6D and Figure S16C and S16D) in agreement with the redundant prognostic value of FoxA1 and ERα [67].

These results are further supported by comparing the PBX1-dependent estrogen induced transcription (Table S2 and Figure S12) against expression profiled from breast tumors using Oncomine (Compendia Bioscience, Ann Arbor, MI). This reveals the strong correlation between PBX1-dependent estrogen target genes and twenty-two expression signatures typical of poor-outcome in breast cancer patients (ex: metastasis, mortality, recurrence and high grade) (p<0.01, O.R. >2) (Figure 6E). In contrast, the FoxA1-dependent estrogen target genes [44] are significantly associated with only one poor-outcome expression signature (mortality) from breast cancer (Figure 6E). Taken together, this suggests that PBX1 drives a very specific transcriptional response underlying progression in ERα-positive breast cancer and reveal the potential prognostic potential for PBX1 within this breast cancer subtype to predict outcome.

## Discussion

**Figure 7:**
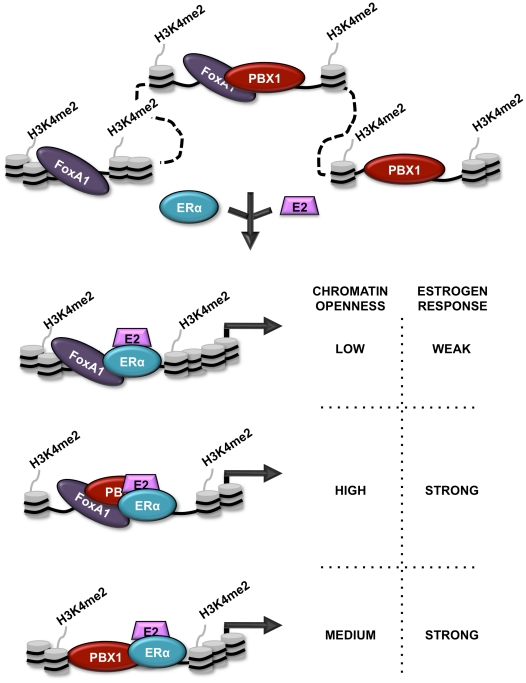
Schematic representation of PBX1 activity in breast cancer. Schematic model depicting the relationship between PBX1, FoxA1 and ERα in breast cancer cells stimulated or not by estrogen/17β-estradiol (E2). Both FoxA1 and PBX1 are bound to the chromatin harboring the H3K4me2 epigenetic signature. They both act independently of each other to open chromatin making specific genomic regions accessible to transcription factors. Stimulation by E2 does not affect their chromatin occupancy but allows ERα recruitment. Importantly, sites of ERα recruitment bound by PBX1, shared or not with FoxA1, are associated with a significant proportion of estrogen responsive genes accounting for a strong estrogen response.

Accurate regulation of complex transcriptional programs is central to normal organ development. This is dependent on several layers of controls including DNA sequence, epigenetic signatures and chromatin structure. However, how these different elements are integrated to generate lineage-specific transcriptional programs and how they are affected in the course of disease development is ill defined. In particular, we still misunderstand how epigenetic signatures and chromatin structure affect the transcriptional response to estrogen stimulation in breast cancer. Here we demonstrate that PBX1 acts as a pioneer factor guiding ERα genomic activity in breast cancer (Figure 7). Indeed, PBX1 translates the H3K4me2-based epigenetic signature to remodel specific genomic domains rendering them accessible for ERα. PBX1 was show to be crucial for histone H4 acetylation [69] and previous reports focusing on the recruitment of MyoD and PDX1 to the chromatin in myeloid and pancreatic islet cells, respectively, were suggestive of the pioneering role of PBX1 [36], [70]. Considering that PBX1 plays a fundamental role in the development of diverse organs [21], [24], [25] and contributes to various types of cancers, namely leukemia, prostate, ovarian and esophageal cancers [26]–[30], its pioneering functions are likely to apply beyond breast cancer. Similarly, the genomic activity of a wide-range of transcription factors including both homeodomain (HOX, MEIS, etc) and non-homeodomain protein (MyoD, GR, TR, etc) is promoted by PBX1 [32], [33], [36], [37], [38], [71], [72]. Hence, PBX1 pioneering functions are expected to affect additional transcriptional programs.

Finally, we reveal that PBX1 and FoxA1 can co-occupy specific genomic regions in breast cancer cells. While co-occupancy of specific genomic region by pioneer factors, such as PU.1 and GATA1 has previously been reported [73], our results demonstrates that this translates into greater chromatin accessibility. Furthermore, we reveal that FoxA1-independent PBX1 bound sites are more accessible than PBX1-independent FoxA1 sites. In agreement, the estrogen induced transcriptional response is preferentially associated with ERα binding at PBX1 or PBX1-FoxA1 shared sites. This also relates to a distinct prognostic value for FoxA1 and PBX1. Indeed, while FoxA1 expression in ERα-positive primary breast tumors does not discriminate their metastasis-free outcome, elevated PBX1 expression has significant prognostic potential towards metastasis. Gene signatures such as the Oncotype DX or MammaPrint have been successfully employed in the clinic to discriminate outcome in breast cancer based mostly on their ability to identify specific breast cancer subtypes [74], [75]. However they do not perform as well when restricted to ERα-positive patients [76], [77]. Our study introduces PBX1 as a potential clinical tool with additive prognostic value to ERα. Indeed, all patients with ERα-positive metastatic breast cancer and half or more of ERα-positive early stage breast cancers develop resistance to endocrine therapies leading to a poor outcome [78]. Hence, it is fascinating to speculate a role for PBX1 in the development of drug resistance in breast cancer.

Taken together, these results reveal the intricate interplay between distinct pioneer factors required for the implementation of specific transcriptional response to estrogen in breast cancer and distinguishes PBX1 as a prognostic marker.

## Materials and Methods

### Motif discovery

FoxA1-independent ERα binding sites across the genome were identified by subtracting the False Discovery Rate (FDR) 20% FoxA1 cistrome from the FDR1% estrogen-induced ERα cistrome from MCF7 breast cancer cells. This was accomplished using the bedfiles that specifies the genomic coordinates for the FoxA1 cistrome called by MAT available through the Cistrome website (http://cistrome.dfci.harvard.edu/ap/) using a cutoff based on the FDR 20% and the bedfile that specifies the genomic coordinates for the ERα cistrome called by MAT using a cutoff based on FDR 1%. These files were loaded on the Cistrome website and the FoxA1 bedfile was subtracted from the ERα bedfile using the “Operate on Genomic Intervals - subtract” [79]. To define the proportion of the ERα cistrome overlapping or not with FoxA1 harboring the PBX1 DNA recognition motif (Transfac M01017) we used the default settings of the “Integrative Analysis – Screen motif” function available on the Cistrome website.

### Correlation analysis

Expression correlation between ERα and PBX1 from the NCI60 cancer cell panel using BioGPS (http://biogps.gnf.org). Expression correlation analysis between ERα and PBX1 in breast cancer cells or primary tumors was achieved using Oncomine (https://www.oncomine.com).

### Overlap analysis and genome structure correction (GSC)

Venn diagrams were generated by defining the proportion of sites shared and unique between different bedfiles using the functions found under “Operate on Genomic Intervals” within the Cistrome website. Overlapping binding sites were defined by having at least one base pair in common. Genome structure correction (GSC) [80] was run to establish the significance of the overlap between datasets. The software was run with the following setting: (region fraction) -R = 0.2, (sub-region fraction) –S = 0.4 and basepair_overlap_marginal (-bm) as statistic text. P values for results presented on Figure S6A and S6B have been corrected using the Bonferroni post-test based on 12 comparisons.

### Immunofluorescence imaging

For immunofluorescence, MCF7 cells were treated as previously described [81]. PBX1 was stained using PBX1 monoclonal antibody (Abnova Corporation). FoxA1 was stained using FoxA1 polyclonal antibody (Abcam). Secondary antibodies Alexa 488 and 555 were purchased from Invitrogen. Digital images were analyzed with ImageJ (http://rsbweb.nih.gov/ij/index.html).

### siRNA Transfection of MCF7 breast cancer cells

MCF7 cells were maintained in phenol red-free medium (Invitrogen) supplemented with 10% CDT-FBS as described previously (Lupien et al. 2008) [6] prior to transfection. Following two days of estrogen starvation cells were transfected with siPBX1 #1 (Darmachon) or siPBX1 #2 (Invitrogen). Small-interfering RNA against Luciferase was used as a negative control [8]. Transfection was performed using Lipofectamine2000 according to manufacturer's instructions (Invitrogen). For cell proliferation assays, cell number or O.D. (450 nm) (WST-1 assay, Takara Bio Inc) was determined every 24 h after estrogen (E2) addition (1×10^−8^ M final). For expression assays, RNA was extracted 3 h following E2 stimulation.

### Microarray

RNA samples from siControl or siPBX1 treated MCF7 in the presence or absence of estrogen were hybridized on HT12 human beads array (Illumina Inc.). Analyses were performed using BRB-Array Tools Version 3.8.1. Raw intensity data were log2 transformed, median normalized and filtered to remove non-detected spots as determined by Illumina Software. The normalization was performed by computing a gene-by-gene difference between each array and the median (reference) array, and subtracting the median difference from the log intensities on that array, so that the gene-by-gene difference between the normalized array and the reference array is zero. Two class non-paired comparison analyses were performed by computing a t-test for each gene using normalized log-intensities. Differentially expressed genes were determined at a significance level of p less than 0.01. A four class ANOVA at p less than 0.01 was also performed to identify genes expressed differentially across the four groups.

Hierarchical clustering was employed using a Euclidean distance measure to generate heat maps for subsets of significant genes using the open source software Cluster/Treeview. The data can be accessed in GEObrowser under superSeries GSE28008

FoxA1 dependent gene-signature was obtained from previously published microarray data [44].

### ChIP and ChIP-reChIP-qPCR

ChIP qPCR was performed as described previously [82]. Antibodies against PBX1 (Abnova) FoxA1, H3K4me2 (Abcam) and ERα (Santa cruz biotechnology) were used in these assays. ChIP–reChIP was performed as described previously [83]. Statistically significant differences were established using a Student's t-test comparison for unpaired data versus an internal negative control. Primer sequences used in this assay are found in Table S3.

### ChIP-seq

ChIP assay were conducted as described above. Library preparation for next-generation sequencing was performed according to manufacturer's instruction starting with 5 ng of material (Illumina Inc.). Single paired libraries were sequenced using the GAIIx (Illumina Inc). Over 28 and 31 million reads were generated through the GAIIx for the PBX1 ChIP and Input samples, respectively. Of those, 88% and 96%, respectively, were aligned to the human reference genome. These reads were aligned using the ELAND software. The MACS peak-calling algorithm was used to call significantly enriched peaks using default settings (P<10^−5^) and specifying the peak size = 200 bp. The data is accessible on the GEObrowser (accession number: PBX1:GSE28008 and H3K4me2:GSE31151).

### FAIRE

FAIRE analysis was performed as previously described [39], [84]. FAIRE-seq data were already published [44].

### Transfection of MCF7 cells

MCF7 cells were maintained in DMEM (Invitrogen) supplemented with 10% FBS as described previously (Lupien et al. 2008) [6] prior to transfection. MCF7 cells were transfected with the pCMX-KDM1construct or the control empty vectors (10 µg per well in 6 well plates) using Lipofectamine 2000 DNA transfection reagent according to the manufacturer's instructions (Invitrogen). ChIP assays against PBX1 were performed 48 h post-transfection.

### Kaplan-Meier curves

Several expression profiles [42], [63], [85], [86], [87], [88], [89], [90], [91] compiled in Oncomine (https://www.oncomine.com) were used to define PBX1 and FoxA1 mRNA expression levels. ERα stratification was based on protein levels provided in each independent expression study employed in this analysis. Samples were ranked according to processed probe signal provided by each independent expression study (Max to Min) and top and bottom 10% were classified as high and low expression respectively. Each sample was then matched with its associated outcome with a 1, 3 and 5 years follow-up provided by each independent study (metastasis-free survival: alive or dead). Statistical analyses were performed using Fisher exact test.

### Transcriptome-based outcome analysis

PBX1-dependent or FoxA1-dependent estrogen (E2) upregulated gene signatures [44] were analyzed against several expression profiles previously shown to be significantly associated with breast cancer outcome using Oncomine. [86], [87], [88], [90], [92], [93], [94], [95], [96], [97], [98], [99], [100], [101], [102], [103] Significant association was established at a pValue of at least <0.01 and an Odds Ratio >2.

## Supporting Information

Figure S1 PBX1 is the main PBX family member expressed in MCF7. (A) The proportion of AR binding sites harboring the PBX1 matrix (Transfac: M01017) is presented. Percentages are calculated based on the previously published 5077 AR binding sites from LNCaP cells treated with DHT for 4 hours (Brown lab) B) PBX family member expression in MCF7 was assessed by RT-qPCR. Data are expressed as percentage of PBX1 in mock induced (O) MCF7 cells. Expression under estrogen/17β-estradiol (E2) is also presented. (p***<0.001).
(TIF)

Figure S2 PBX1 suppresses estrogen-induced proliferation. (A) MCF7 cells were stimulated with estrogen/17β-estradiol (E2) or control (O) with or without PBX1 silencing via siRNA and cell were counted after 2 days and compared to control (siCTRL) (p*<0.05, **<0.01) (B) Comparison of cell number in MCF7 cells treated with siPBX1 vs siCTR in a estrogen-deprived media (O).
(TIF)

Figure S3 PBX1 and FoxA1 partially co-localize in MCF7 cells nucleus. (A) Protein localization was analyzed after PBX1 and FoxA1 staining via digital imaging. (B) Same as A but with the added Z-axis represent staining intensity.
(TIF)

Figure S4 ERα recruitment is specifically disrupted at PBX1 bound sites. (A) CEAS analysis demonstrate genomic distribution of PBX1 binding in MCF7 breast cancer cells (B) ChIP-qPCR assays against PBX1 were conducted to validate PBX1 ChIP-seq results in MCF7 breast cancer cells treated with estrogen/17β-estradiol (E2) or control (O). (C) ChIP-qPCR assays in MCF7 cells depleted of estrogen against PBX1 demonstrate that it is not present at the tested ERα binding sites while it is efficiently detected at the positive control (pos. CTRL) site.
(TIF)

Figure S5 ChIP-seq tracks. Raw massively parallel sequencing (WIG lines) and called peaks (BED lines) derived signal for ERα (estrogen stimulated), PBX1 (full media), FoxA1 (full media), FAIRE (untreated) and H3K4me2 (untreated) signal from MCF7 at representative genomic locations were obtained using the integrated genomic viewer (IGV 2.0). Boxes were used to underscore the primers used in this study.
(TIF)

Figure S6 ChIP-seq tracks. Raw massively parallel sequencing (WIG lines) and called peaks (BED lines) derived signal for ERα (estrogen stimulated), PBX1 (full media), FoxA1 (full media), FAIRE (untreated) and H3K4me2 (untreated) signal from MCF7 at representative genomic locations were obtained using the integrated genomic viewer (IGV 2.0). Boxes were used to underscore the primers used in this study.
(TIF)

Figure S7 ChIP-seq tracks. Raw massively parallel sequencing (WIG lines) and called peaks (BED lines) derived signal for ERα (estrogen stimulated), PBX1 (full media), FoxA1 (full media), FAIRE (untreated) and H3K4me2 (untreated) signal from MCF7 at representative genomic locations were obtained using the integrated genomic viewer (IGV 2.0). Boxes were used to underscore the primers used in this study.
(TIF)

Figure S8 ChIP-seq tracks. Raw massively parallel sequencing (WIG lines) and called peaks (BED lines) derived signal for ERα (estrogen stimulated), PBX1 (full media), FoxA1 (full media), FAIRE (untreated) and H3K4me2 (untreated) signal from MCF7 at representative genomic locations were obtained using the integrated genomic viewer (IGV 2.0). Boxes were used to underscore the primers used in this study.
(TIF)

Figure S9 Cistromes intersections. GSC analysis of various cistromes (ERα, FoxA1, and AR) against PBX1.
(TIF)

Figure S10 Cistromes intersections. GSC analysis of PBX1 cistrome against ERα, FoxA1 and AR cistromes.
(TIF)

Figure S11 PBX1 and FoxA1 co-localize on the chromatin. ChIP-reChIP assay demonstrates that PBX1 and FoxA1 can co-bind the same DNA sites in MCF7 cells in absence of estrogen (O). Matched IgG were used in the reChIP as negative control.
(TIF)

Figure S12 Expression profile defines the PBX1-dependent estrogen regulated genes in MCF7 breast cancer cells. Heatmap displayed as a ratio between estrogen/17β-estradiol (E2) and control (O) treated cells in MCF7 breast cancer cells depleted or not of PBX1 by siRNA. Yellow relates to E2 induction while blue relates to E2 repression.
(TIF)

Figure S13 PBX1 and FoxA1 silencing selectively impairs E2 response. Histogram of the data presented in Figure 3D. Asterisks represent significant difference determined by one-way ANOVA analysis vs. siCTRL (p<0.05).
(TIF)

Figure S14 PBX1 silencing removes PBX1 from the chromatin. (A) Percentage of number of sites overlapping with peaks of FAIRE signal called by the MACS peak-calling algorithm. This demonstrates that FAIRE is significantly associated with PBX1-FoxA1 shared sites versus PBX1 of FoxA1 unique sites. (B) MCF7 cells were cultured in estrogen-free media and treated with siPBX1. ChIP-qPCR assays against PBX1 were performed in siPBX1 and siCTRL transfected cells. Values are expressed as percentage of reduction of PBX1 presence on the chromatin in siPBX1 versus siCTRL transfected cells (p*<0.05).
(TIF)

Figure S15 PBX1 silencing does not alter the epigenetic signature H3K4me2. (A) Depleting MCF7 cells of PBX1 via siRNA does not have a significant effect on H3K4me2 levels as determined by ChIP-qPCR in absence of estrogen. Relative fold enrichment is expressed as fold over negative internal control. (B) PBX1 silencing does not alter the expression of the H3K4me2 specific de-methylases KDM1 regardless of estrogen (E2) or control (O) treatment.
(TIF)

Figure S16 PBX1 prognostic potential in breast cancer. (A–B) Kaplan-Meier curve were generated using KMplotter splitting patients using the upper quartile. ERα and FoxA1can significantly predict metastasis development in breast cancer subtype. Beeswarm graphs are used to plot probe distribution. (C–D) Kaplan-Meier curve were generated as in A–B limiting the analysis to ERα-positive breast cancer subtype as defined by pathological staining. PBX1 can significantly predict metastasis development in ERα-positive breast cancer subtype.
(TIF)

Figure S17 Expression levels for FoxA1 and PBX1 across primary breast tumors. The average level of FoxA1 (left panel) and PBX1 (right panel) mRNA levels in primary breast tumors compiled for to generate the Kaplan-Meier curves (Figure 6) are presented. The difference in FoxA1 mRNA expression between the high and low FoxA1 expressers is greatest across all breast cancer subtypes as opposed to the ERα-positive breast cancer subtype. The difference in PBX1 mRNA expression between the high and low PBX1 expressers remains the same when assessed across all breast cancer subtypes or the ERα-positive breast cancer subtype.
(TIF)

Table S1 Gene coexpressed in the NCI60 cancer cell lines with PBX1.
(XLS)

Table S2 Microarray analysis of genes significantly changed between control and estradiol stimulation (pvalue<0.01).
(XLS)

Table S3 List of primers used in the study.
(XLS)
