# Supplementary figures and images for: PBX1 Genomic Pioneer Function Drives ERα Signaling Underlying Progression in Breast Cancer
Source: PLoS Genet. 2011 Nov 17;7(11):e1002368. doi: 10.1371/journal.pgen.1002368 (PMC3219601; doi:10.1371/journal.pgen.1002368)

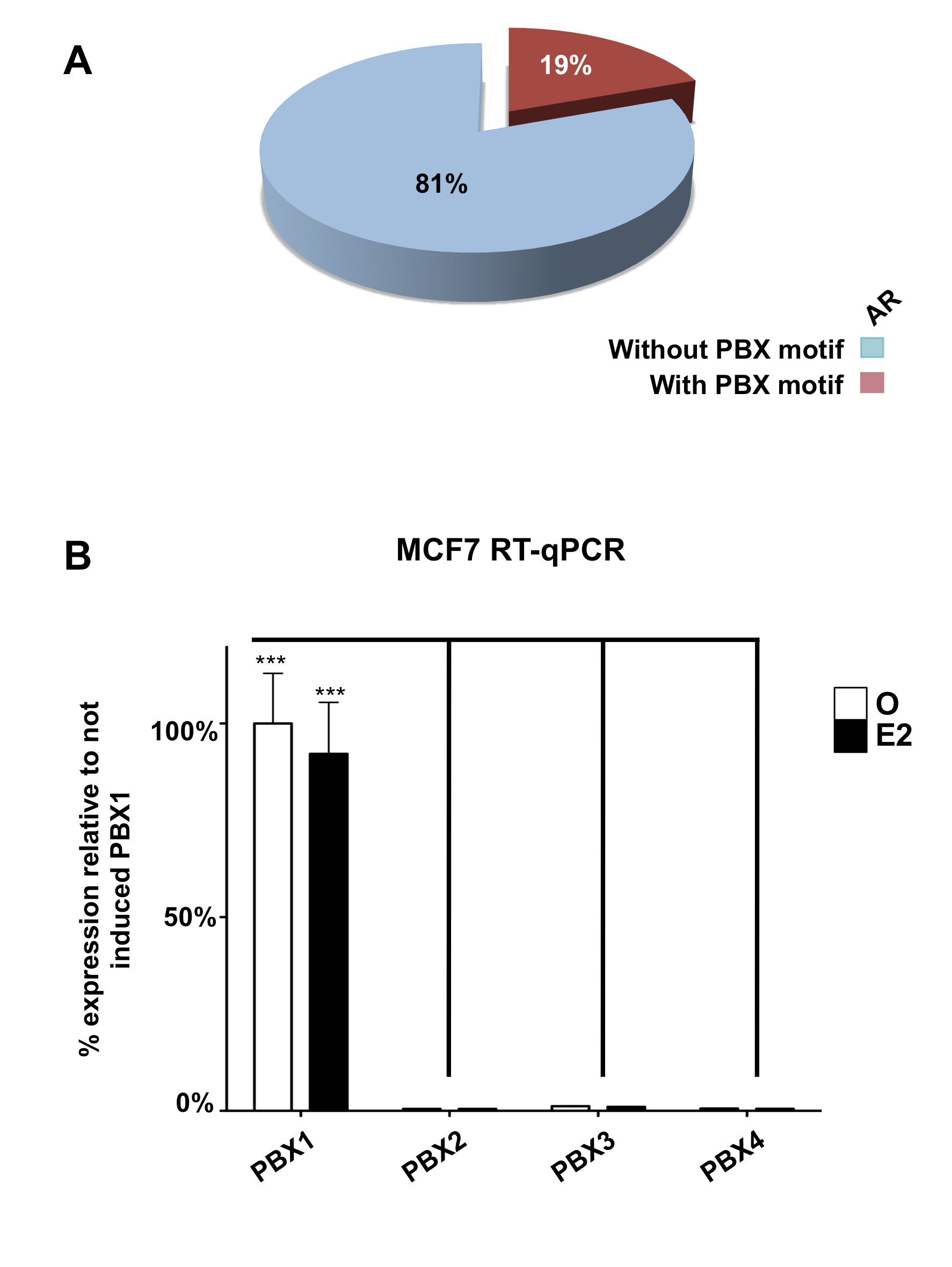

Supplement: Figure S1 — PBX1 is the main PBX family member expressed in MCF7. (A) The proportion of AR binding sites harboring the PBX1 matrix (Transfac: M01017) is presented. Percentages are calculated based on the previously published 5077 AR binding sites from LNCaP cells treated with DHT for 4 hours (Brown lab) B) PBX family member expression in MCF7 was assessed by RT-qPCR. Data are expressed as percentage of PBX1 in mock induced (O) MCF7 cells. Expression under estrogen/17β-estradiol (E2) is also presented. (p***<0.001). (TIF) [file pgen.1002368.s001.tif]

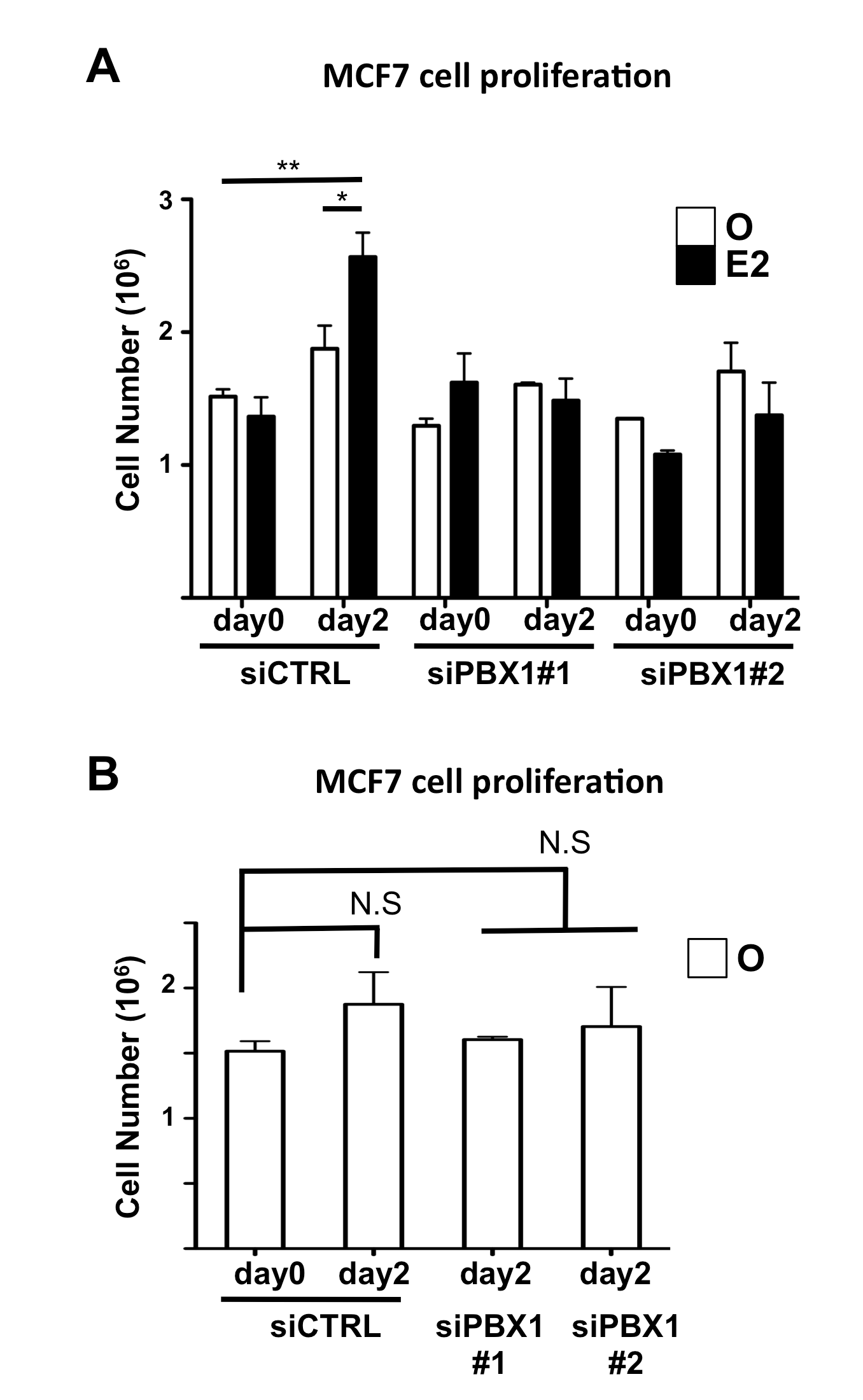

Supplement: Figure S2 — PBX1 suppresses estrogen-induced proliferation. (A) MCF7 cells were stimulated with estrogen/17β-estradiol (E2) or control (O) with or without PBX1 silencing via siRNA and cell were counted after 2 days and compared to control (siCTRL) (p*<0.05, **<0.01) (B) Comparison of cell number in MCF7 cells treated with siPBX1 vs siCTR in a estrogen-deprived media (O). (TIF) [file pgen.1002368.s002.tif]

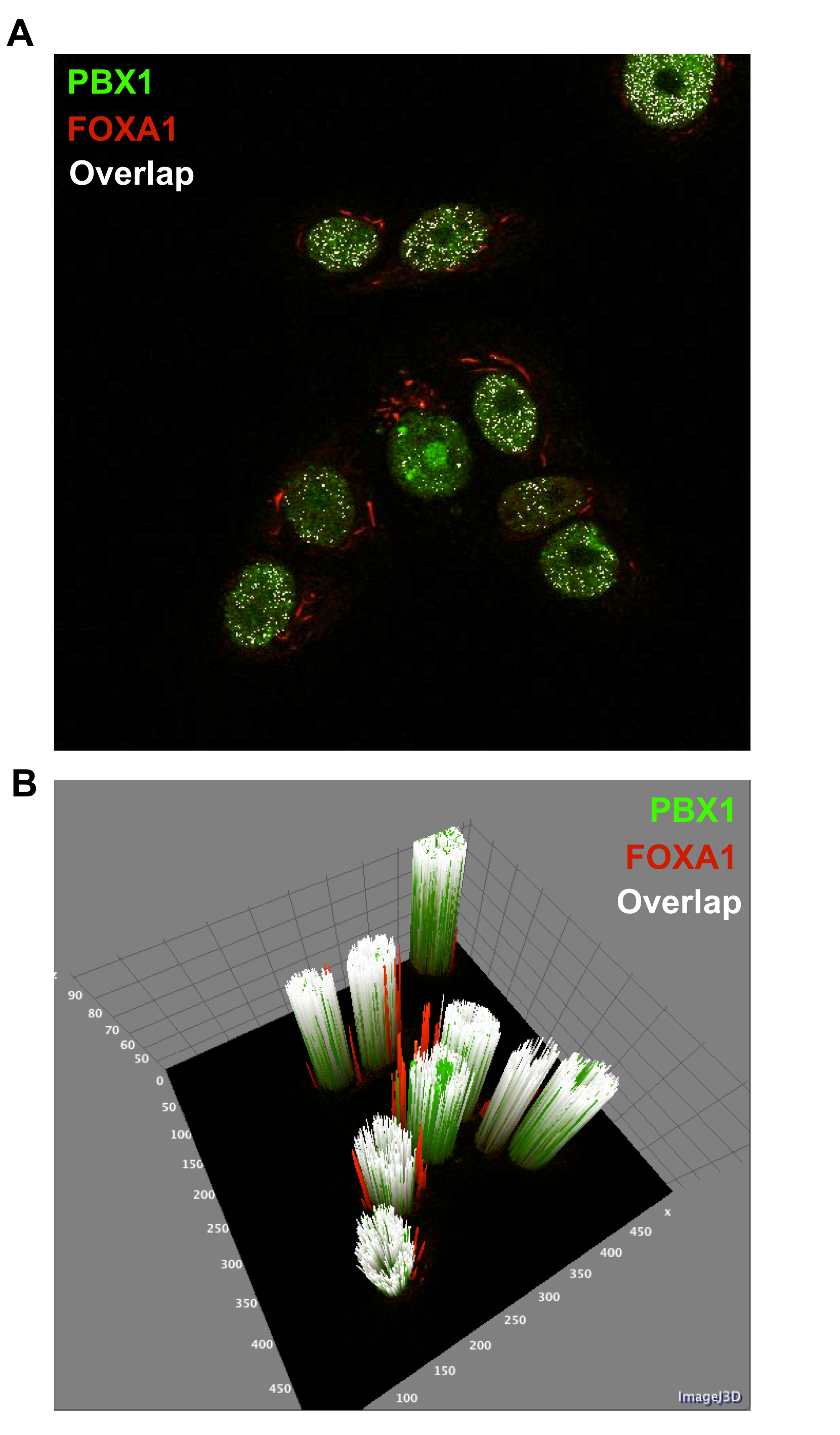

Supplement: Figure S3 — PBX1 and FoxA1 partially co-localize in MCF7 cells nucleus. (A) Protein localization was analyzed after PBX1 and FoxA1 staining via digital imaging. (B) Same as A but with the added Z-axis represent staining intensity. (TIF) [file pgen.1002368.s003.tif]

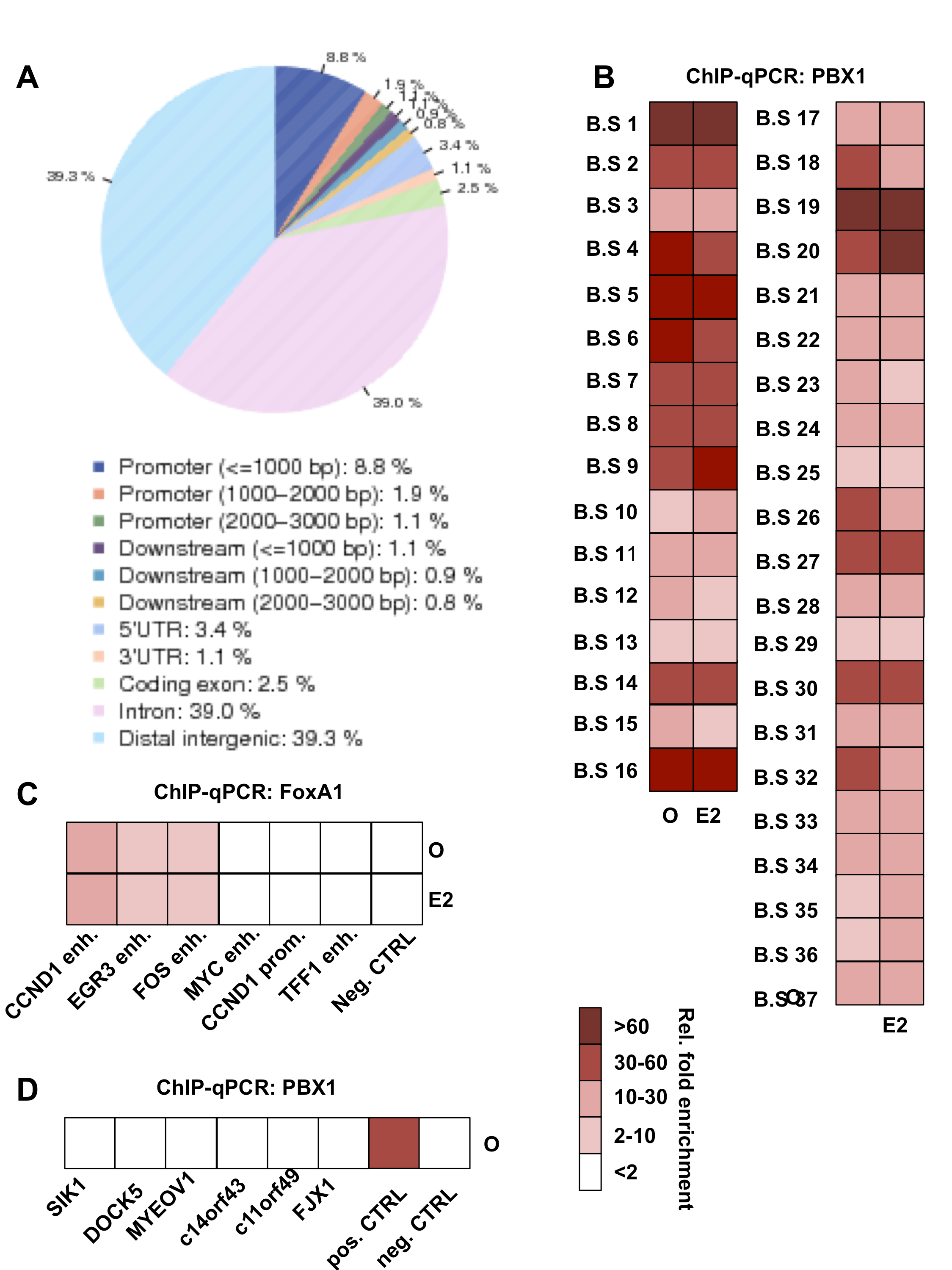

Supplement: Figure S4 — ERα recruitment is specifically disrupted at PBX1 bound sites. (A) CEAS analysis demonstrate genomic distribution of PBX1 binding in MCF7 breast cancer cells (B) ChIP-qPCR assays against PBX1 were conducted to validate PBX1 ChIP-seq results in MCF7 breast cancer cells treated with estrogen/17β-estradiol (E2) or control (O). (C) ChIP-qPCR assays in MCF7 cells depleted of estrogen against PBX1 demonstrate that it is not present at the tested ERα binding sites while it is efficiently detected at the positive control (pos. CTRL) site. (TIF) [file pgen.1002368.s004.tif]

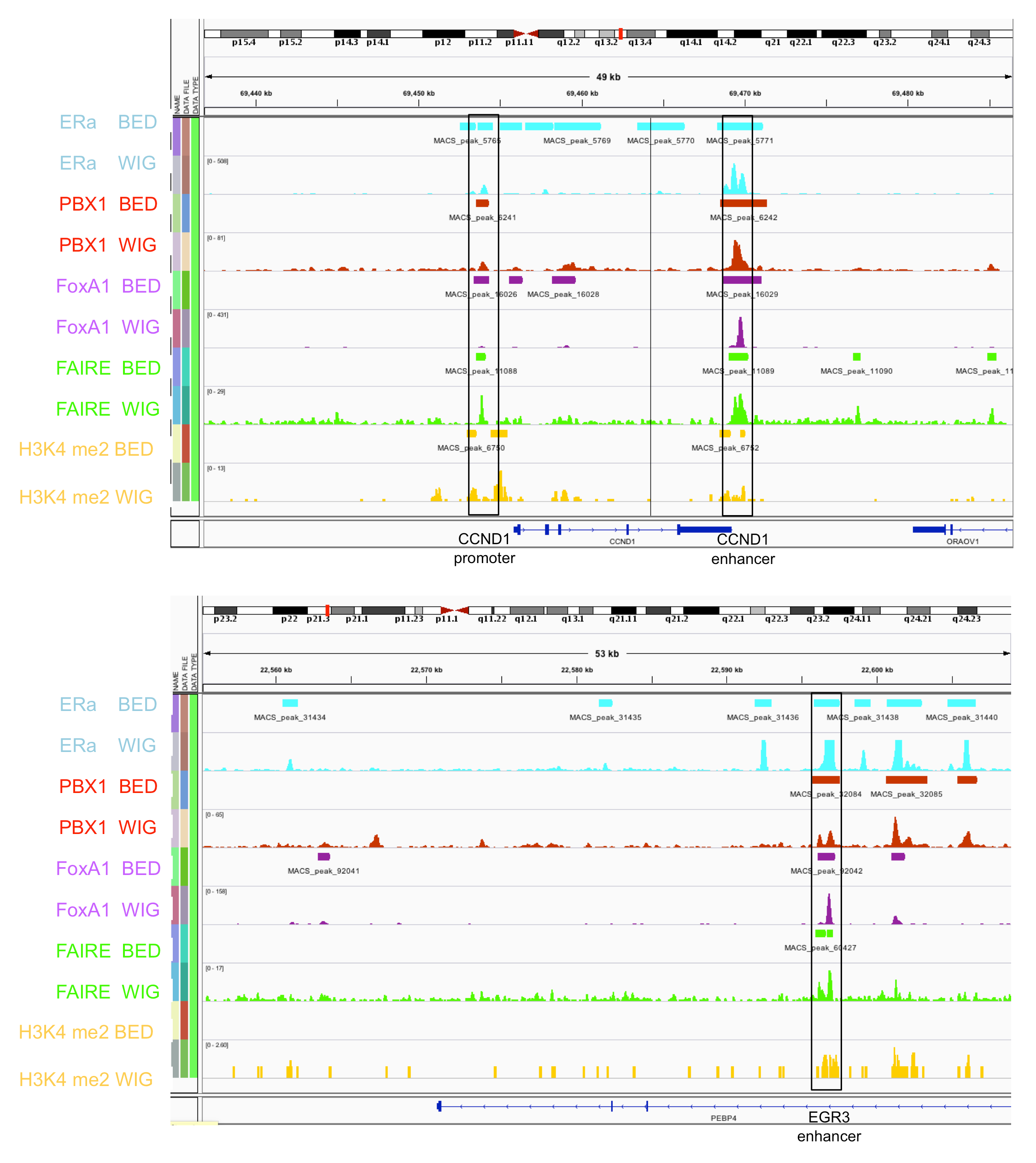

Supplement: Figure S5 — ChIP-seq tracks. Raw massively parallel sequencing (WIG lines) and called peaks (BED lines) derived signal for ERα (estrogen stimulated), PBX1 (full media), FoxA1 (full media), FAIRE (untreated) and H3K4me2 (untreated) signal from MCF7 at representative genomic locations were obtained using the integrated genomic viewer (IGV 2.0). Boxes were used to underscore the primers used in this study. (TIF) [file pgen.1002368.s005.tif]

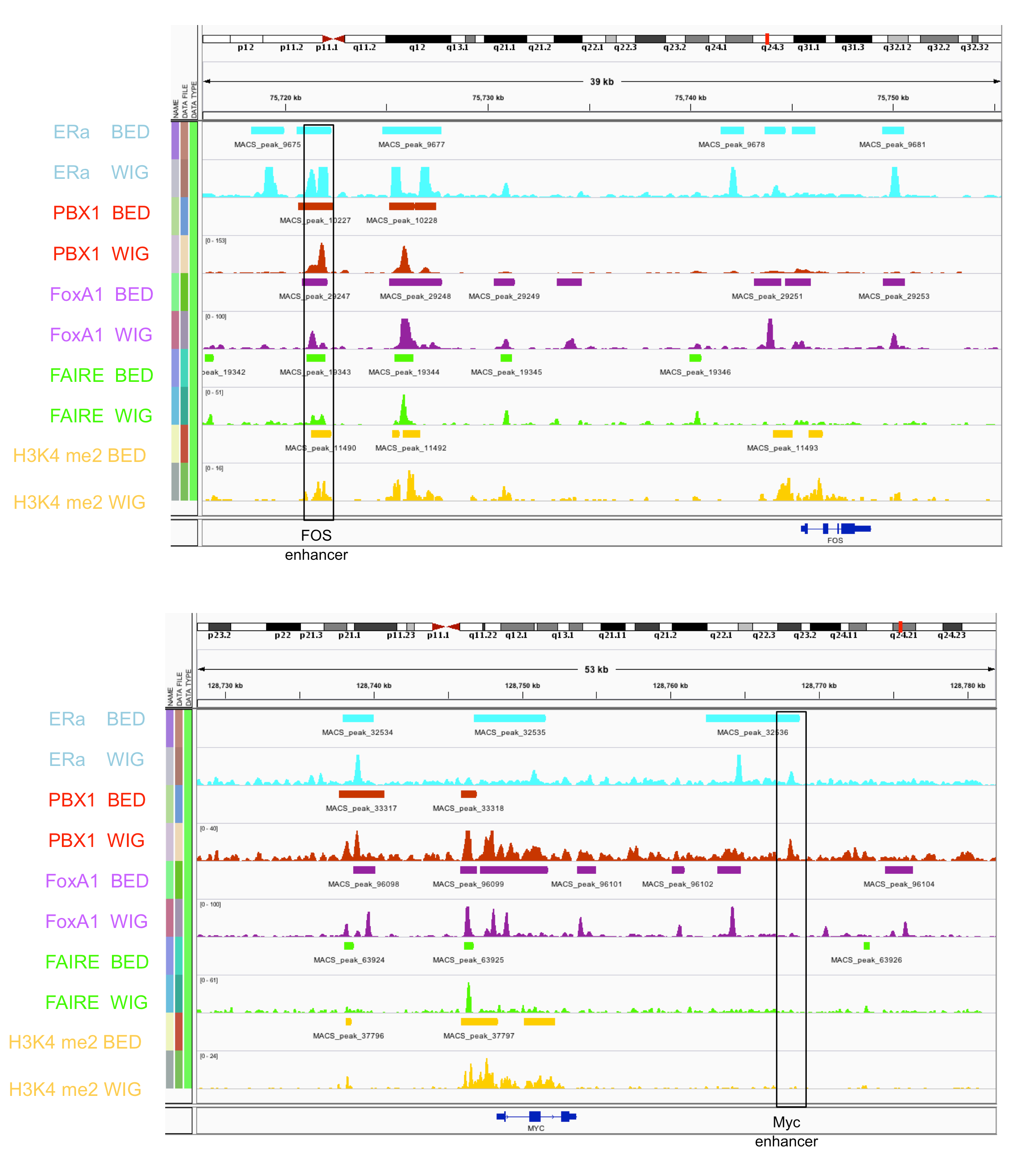

Supplement: Figure S6 — ChIP-seq tracks. Raw massively parallel sequencing (WIG lines) and called peaks (BED lines) derived signal for ERα (estrogen stimulated), PBX1 (full media), FoxA1 (full media), FAIRE (untreated) and H3K4me2 (untreated) signal from MCF7 at representative genomic locations were obtained using the integrated genomic viewer (IGV 2.0). Boxes were used to underscore the primers used in this study. (TIF) [file pgen.1002368.s006.tif]

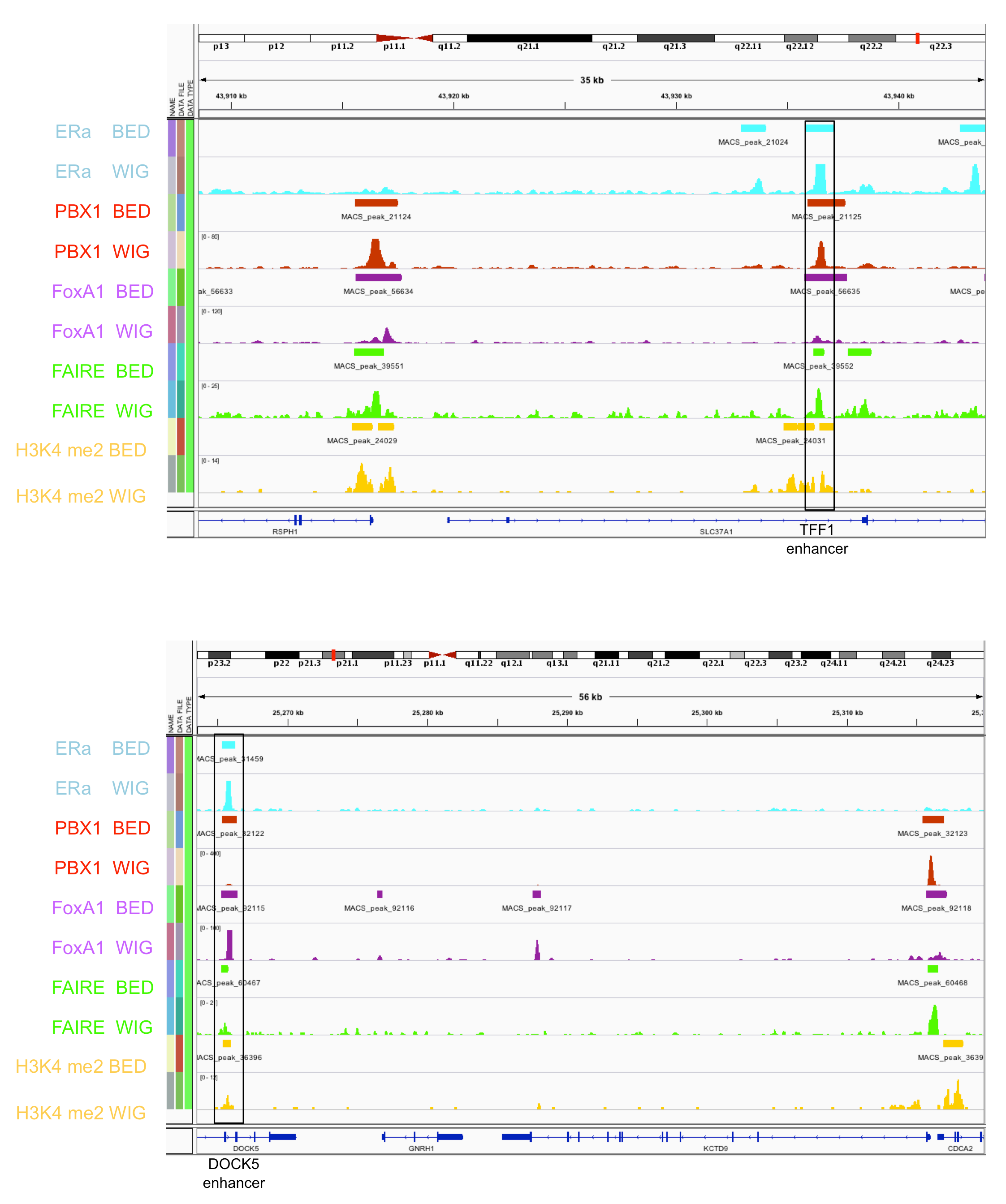

Supplement: Figure S7 — ChIP-seq tracks. Raw massively parallel sequencing (WIG lines) and called peaks (BED lines) derived signal for ERα (estrogen stimulated), PBX1 (full media), FoxA1 (full media), FAIRE (untreated) and H3K4me2 (untreated) signal from MCF7 at representative genomic locations were obtained using the integrated genomic viewer (IGV 2.0). Boxes were used to underscore the primers used in this study. (TIF) [file pgen.1002368.s007.tif]

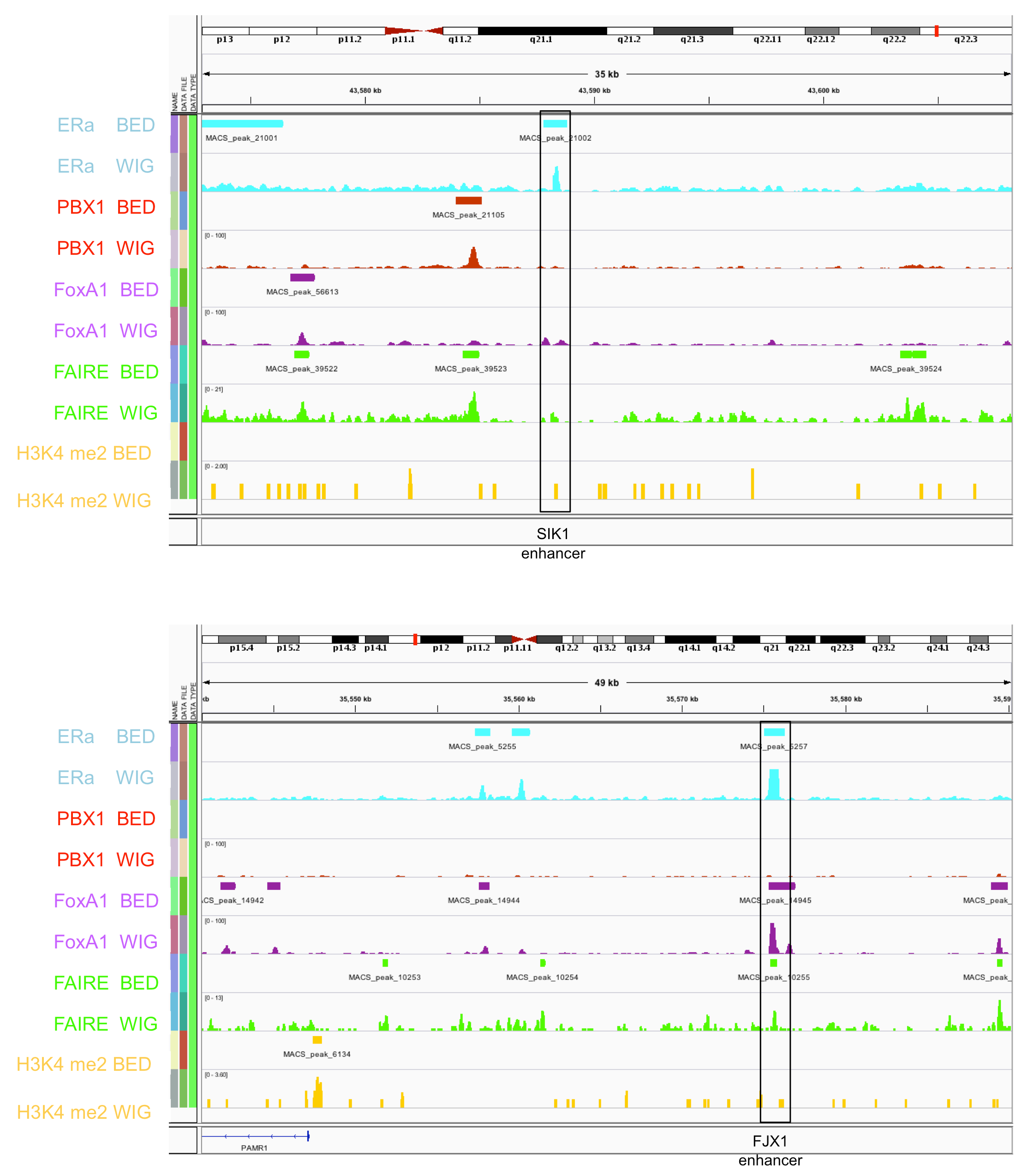

Supplement: Figure S8 — ChIP-seq tracks. Raw massively parallel sequencing (WIG lines) and called peaks (BED lines) derived signal for ERα (estrogen stimulated), PBX1 (full media), FoxA1 (full media), FAIRE (untreated) and H3K4me2 (untreated) signal from MCF7 at representative genomic locations were obtained using the integrated genomic viewer (IGV 2.0). Boxes were used to underscore the primers used in this study. (TIF) [file pgen.1002368.s008.tif]

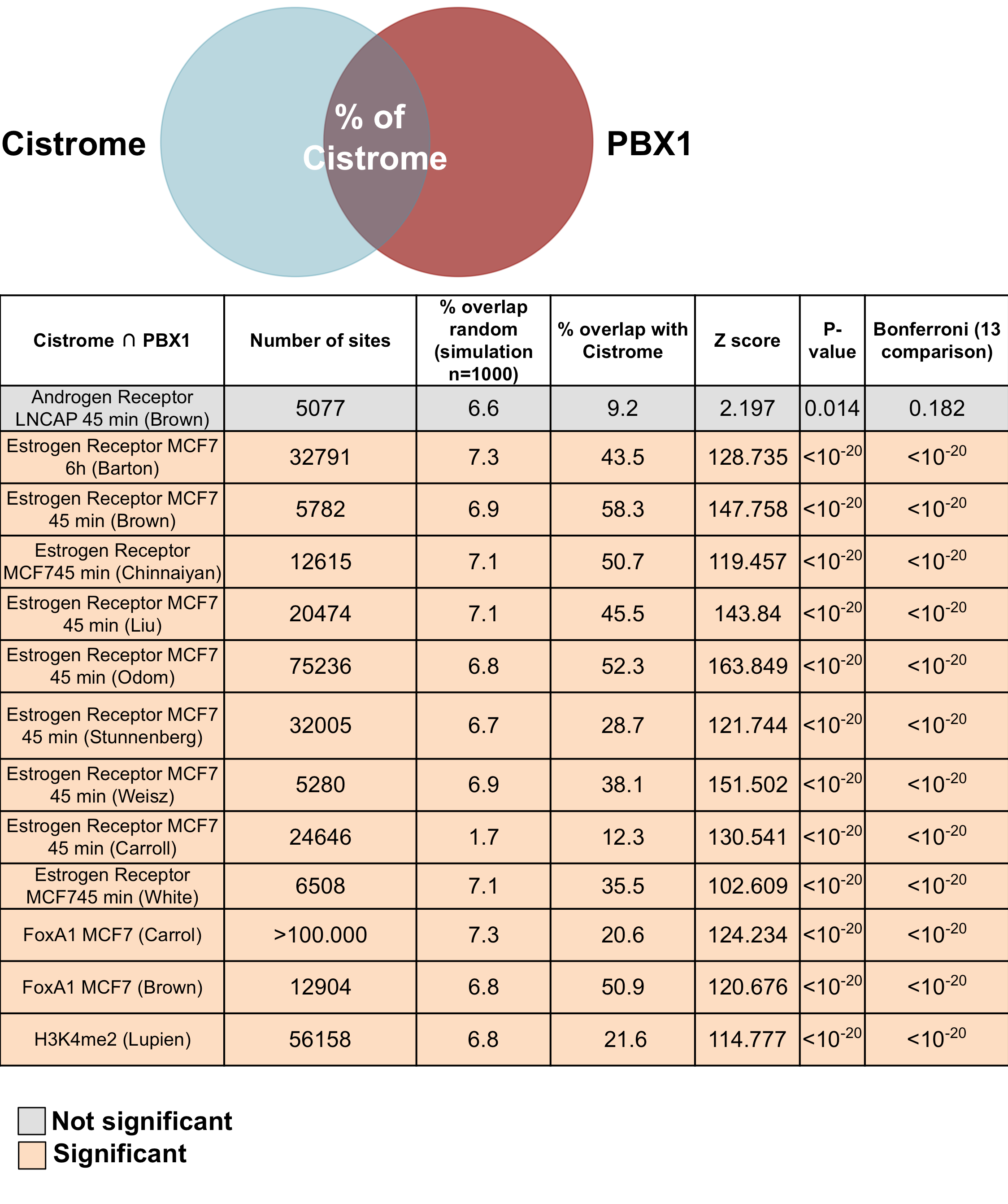

Supplement: Figure S9 — Cistromes intersections. GSC analysis of various cistromes (ERα, FoxA1, and AR) against PBX1. (TIF) [file pgen.1002368.s009.tif]

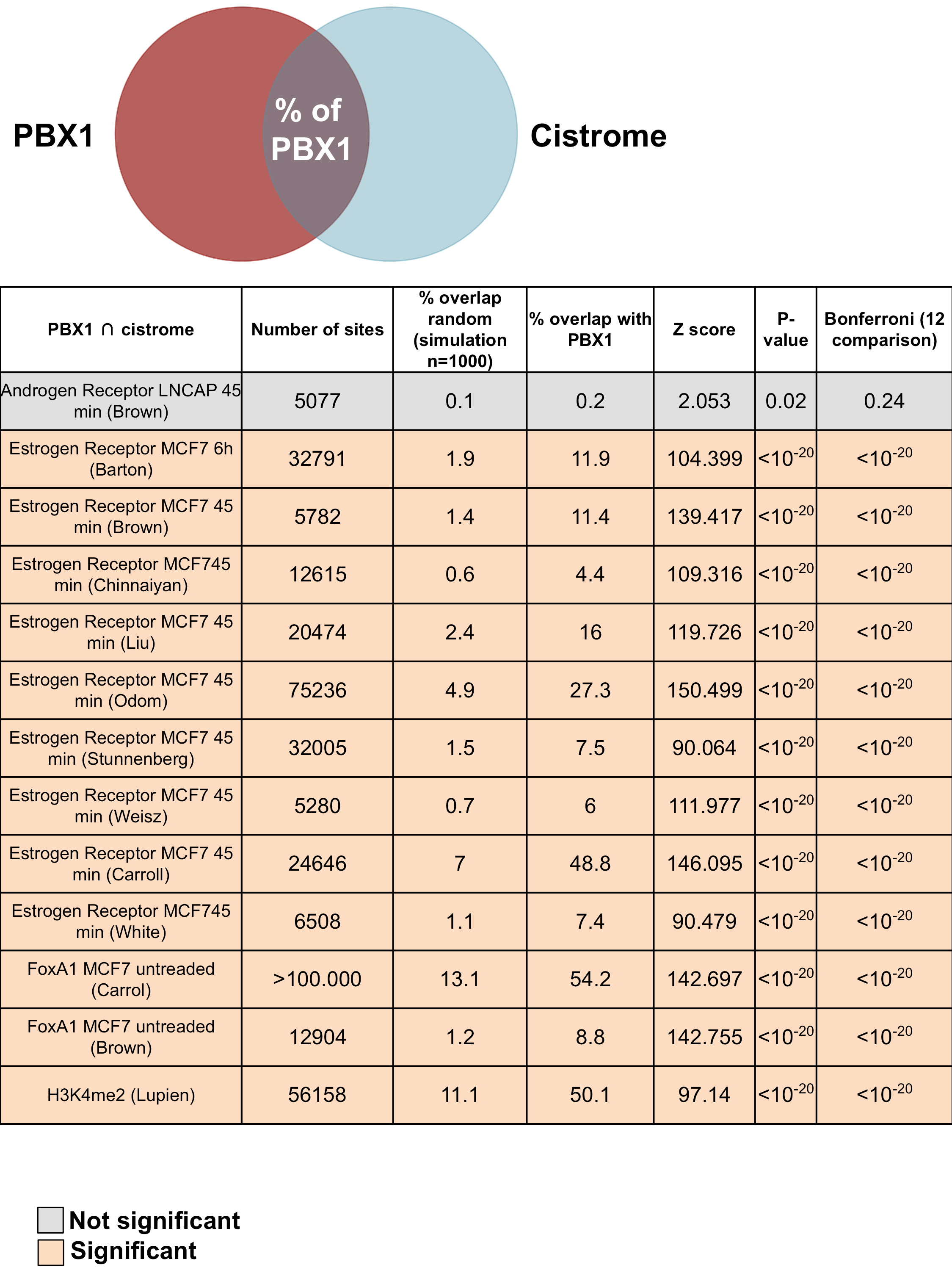

Supplement: Figure S10 — Cistromes intersections. GSC analysis of PBX1 cistrome against ERα, FoxA1 and AR cistromes. (TIF) [file pgen.1002368.s010.tif]

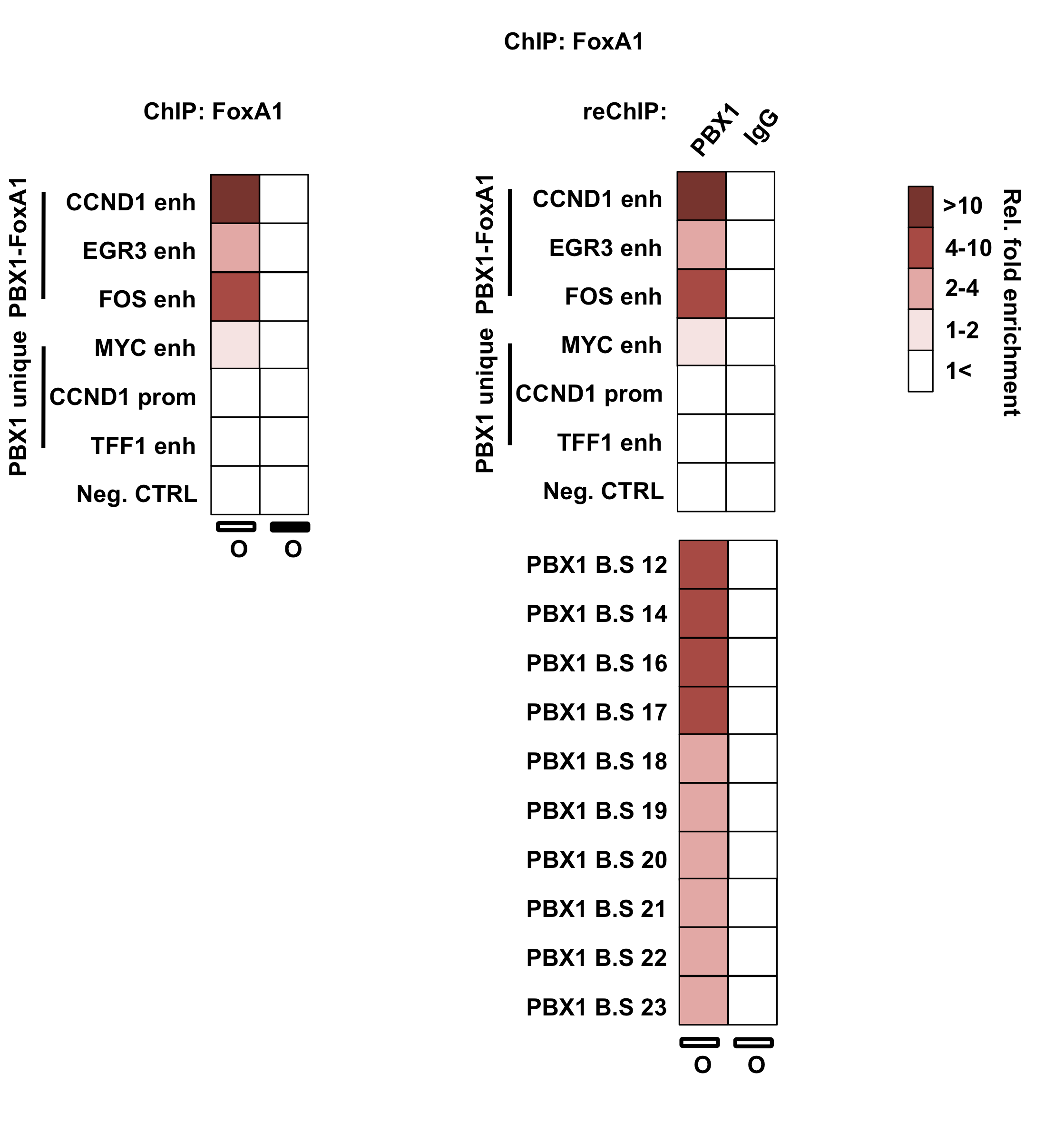

Supplement: Figure S11 — PBX1 and FoxA1 co-localize on the chromatin. ChIP-reChIP assay demonstrates that PBX1 and FoxA1 can co-bind the same DNA sites in MCF7 cells in absence of estrogen (O). Matched IgG were used in the reChIP as negative control. (TIF) [file pgen.1002368.s011.tif]

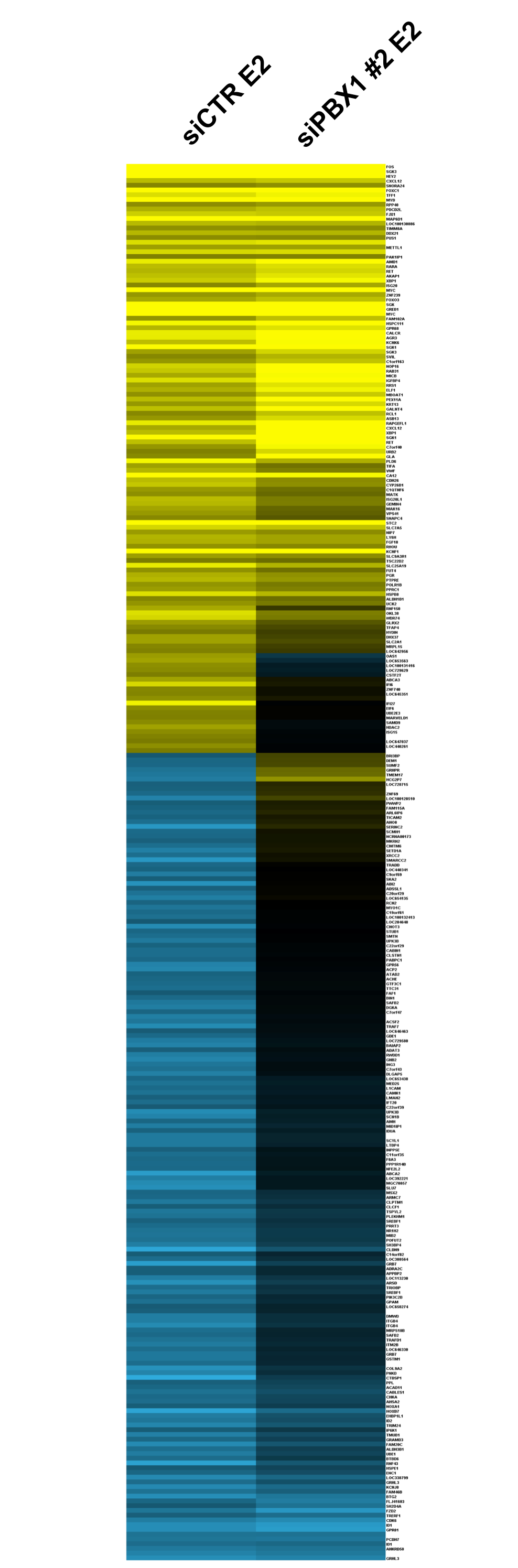

Supplement: Figure S12 — Expression profile defines the PBX1-dependent estrogen regulated genes in MCF7 breast cancer cells. Heatmap displayed as a ratio between estrogen/17β-estradiol (E2) and control (O) treated cells in MCF7 breast cancer cells depleted or not of PBX1 by siRNA. Yellow relates to E2 induction while blue relates to E2 repression. (TIF) [file pgen.1002368.s012.tif]

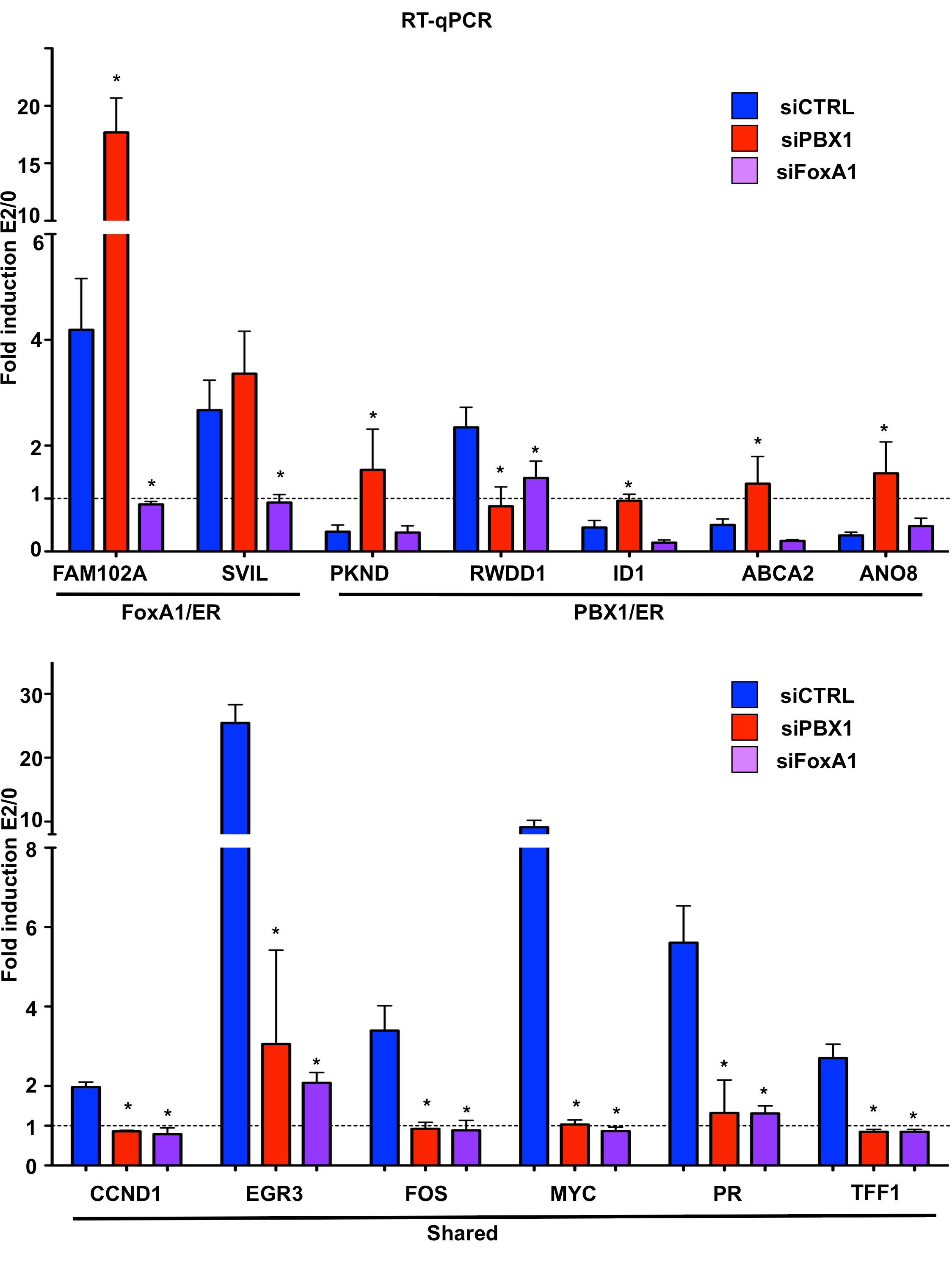

Supplement: Figure S13 — PBX1 and FoxA1 silencing selectively impairs E2 response. Histogram of the data presented in Figure 3D. Asterisks represent significant difference determined by one-way ANOVA analysis vs. siCTRL (p<0.05). (TIF) [file pgen.1002368.s013.tif]

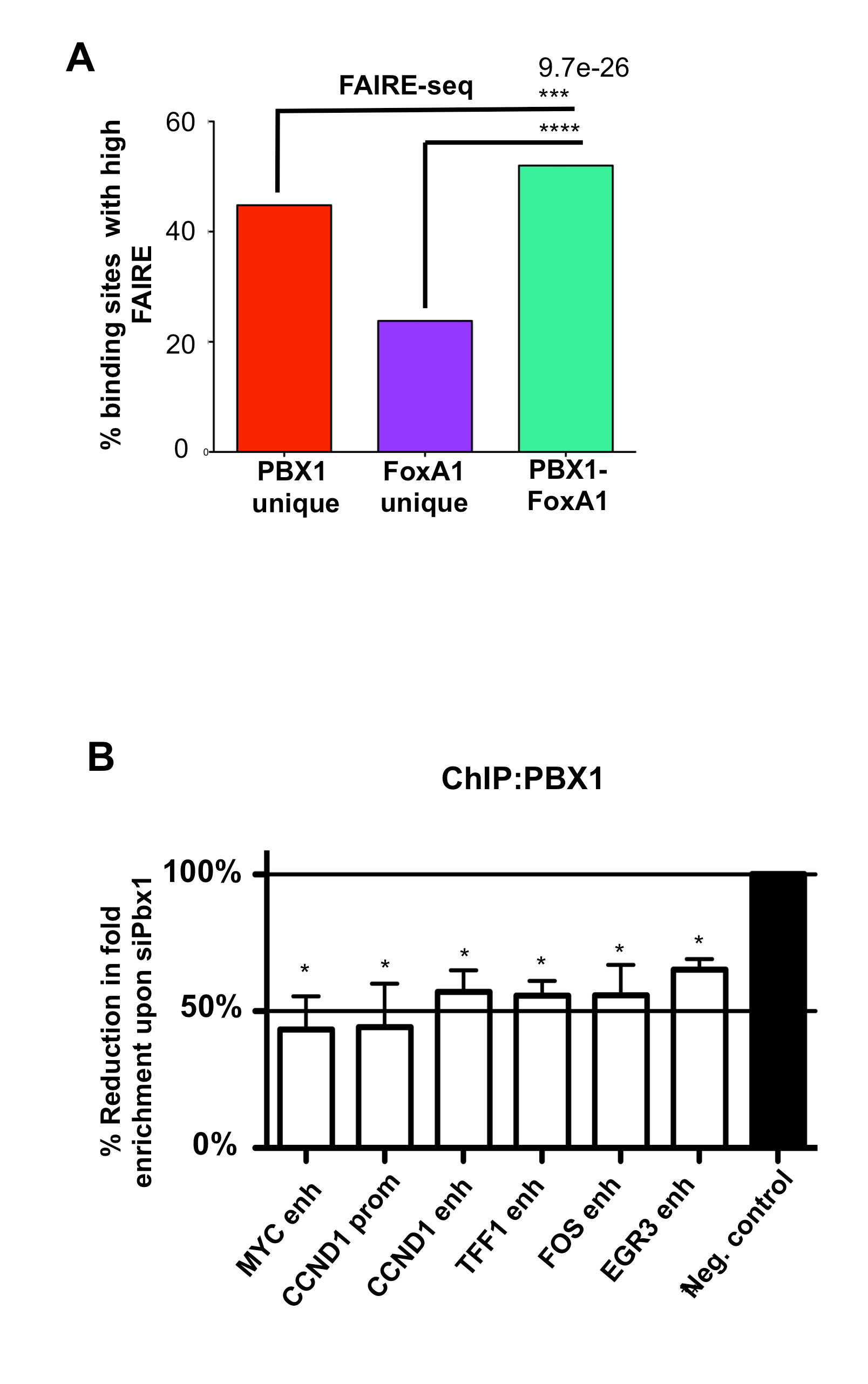

Supplement: Figure S14 — PBX1 silencing removes PBX1 from the chromatin. (A) Percentage of number of sites overlapping with peaks of FAIRE signal called by the MACS peak-calling algorithm. This demonstrates that FAIRE is significantly associated with PBX1-FoxA1 shared sites versus PBX1 of FoxA1 unique sites. (B) MCF7 cells were cultured in estrogen-free media and treated with siPBX1. ChIP-qPCR assays against PBX1 were performed in siPBX1 and siCTRL transfected cells. Values are expressed as percentage of reduction of PBX1 presence on the chromatin in siPBX1 versus siCTRL transfected cells (p*<0.05). (TIF) [file pgen.1002368.s014.tif]

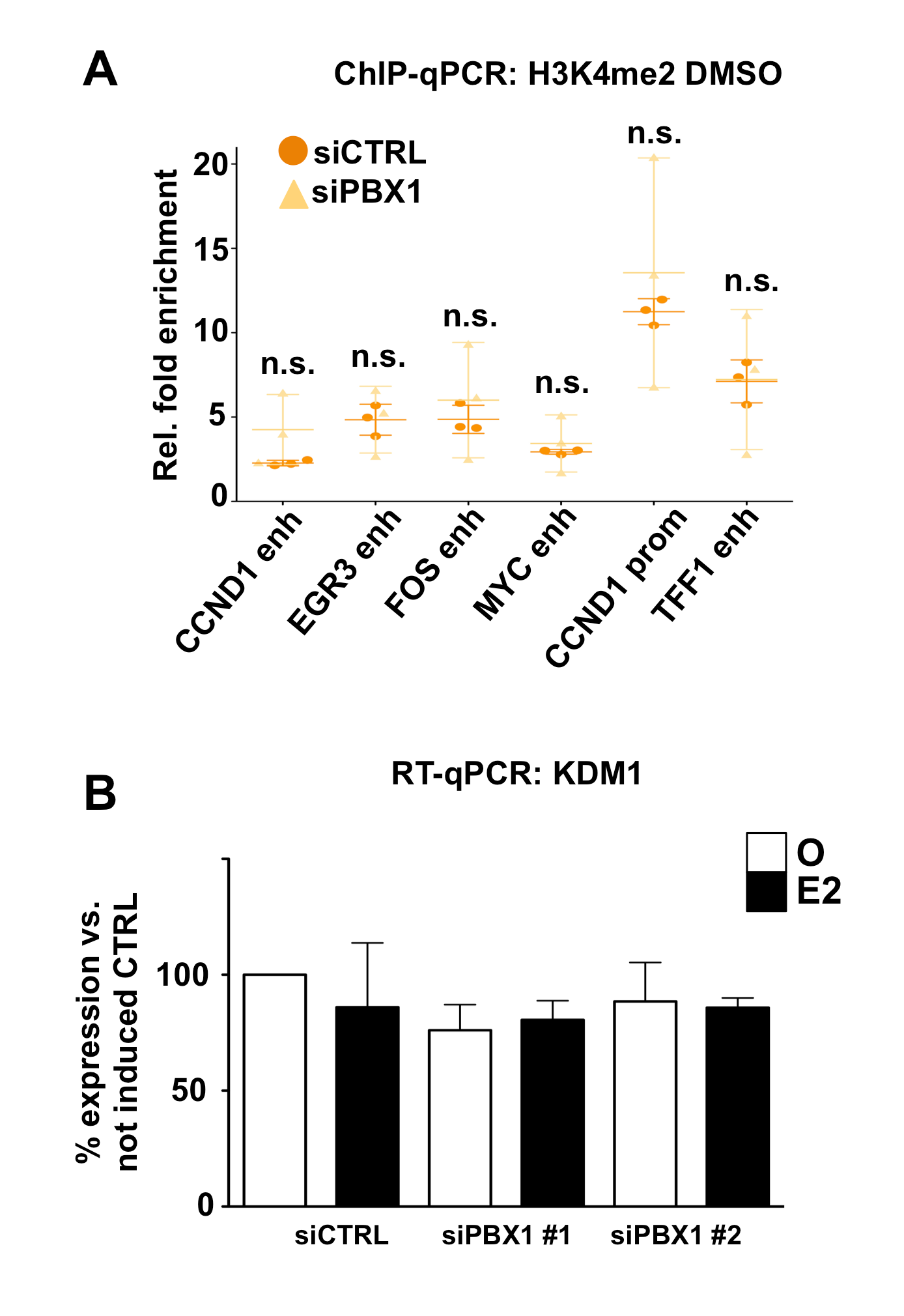

Supplement: Figure S15 — PBX1 silencing does not alter the epigenetic signature H3K4me2. (A) Depleting MCF7 cells of PBX1 via siRNA does not have a significant effect on H3K4me2 levels as determined by ChIP-qPCR in absence of estrogen. Relative fold enrichment is expressed as fold over negative internal control. (B) PBX1 silencing does not alter the expression of the H3K4me2 specific de-methylases KDM1 regardless of estrogen (E2) or control (O) treatment. (TIF) [file pgen.1002368.s015.tif]

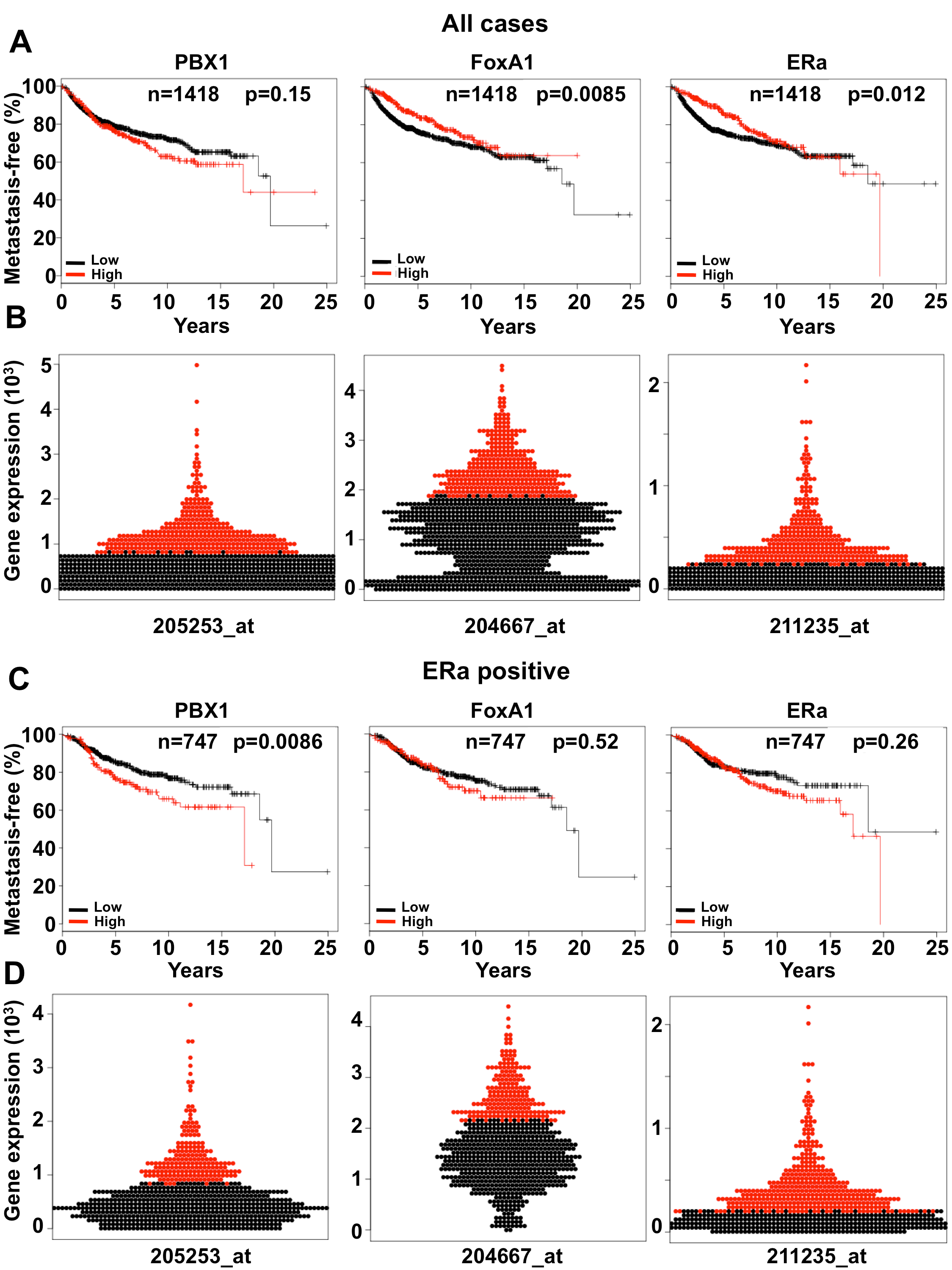

Supplement: Figure S16 — PBX1 prognostic potential in breast cancer. (A–B) Kaplan-Meier curve were generated using KMplotter splitting patients using the upper quartile. ERα and FoxA1can significantly predict metastasis development in breast cancer subtype. Beeswarm graphs are used to plot probe distribution. (C–D) Kaplan-Meier curve were generated as in A–B limiting the analysis to ERα-positive breast cancer subtype as defined by pathological staining. PBX1 can significantly predict metastasis development in ERα-positive breast cancer subtype. (TIF) [file pgen.1002368.s016.tif]

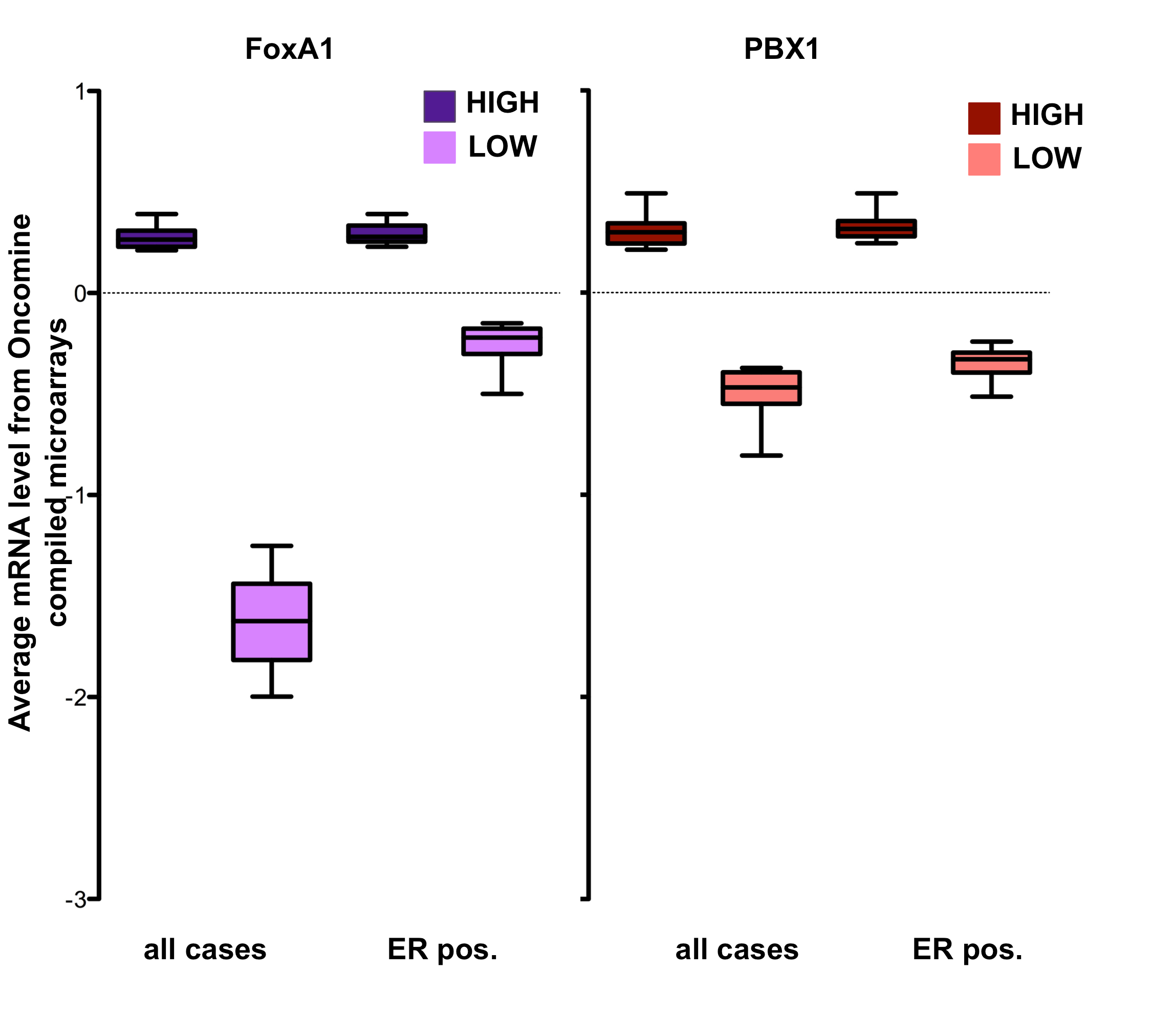

Supplement: Figure S17 — Expression levels for FoxA1 and PBX1 across primary breast tumors. The average level of FoxA1 (left panel) and PBX1 (right panel) mRNA levels in primary breast tumors compiled for to generate the Kaplan-Meier curves (Figure 6) are presented. The difference in FoxA1 mRNA expression between the high and low FoxA1 expressers is greatest across all breast cancer subtypes as opposed to the ERα-positive breast cancer subtype. The difference in PBX1 mRNA expression between the high and low PBX1 expressers remains the same when assessed across all breast cancer subtypes or the ERα-positive breast cancer subtype. (TIF) [file pgen.1002368.s017.tif]
